# Three-Dimensional Functionalization of Macrocycles: Porphyrin and Polycyclic Carbon Cage Hybrid

**DOI:** 10.1021/acs.orglett.6c01942

**Published:** 2026-06-08

**Authors:** Katarzyna Stasiak, Katarzyna Ślepokura, Aleksandra Szumańska, Michał J. Białek, Lechosław Latos-Grażyński, Anna Berlicka

**Affiliations:** Department of Chemistry, 49572 University of Wrocław, 14 F. Joliot-Curie, 50-383 Wrocław, Poland

## Abstract

A three-dimensional functionalization strategy for porphyrins affords a hybrid architecture integrating a porphyrinic structural motif with a rigid, chiral bisnorditwistane cage. The resulting system combines a sterically demanding carbon framework with a planar triheterocyclic fragment. The macrocycle exhibits a well-defined two-step protonation behavior.

## Graphical abstract

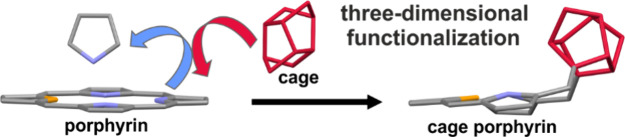

The design of new porphyrinic macrocycles provides a convenient method for synthesizing compounds with specific geometries, coordination properties, spectroscopic properties, and reactivity. In recent years, there has been dynamic development in the chemistry of carbaporphyrins, i.e., porphyrins modified in the coordination core by replacing nitrogen atom(s) with carbon atom(s). Carbaporphyrins can be synthesized by incorporating carbocyclic units, such as cyclopentadiene, benzene, or azulene, into the porphyrinoid framework.^1–3^ Alternatively, fusion inclusion of polycyclic aromatic hydrocarbons (naphthalene, anthracene, and phenanthrene) affords a distinct class of carbaporphyrinoids known as aceneporphyrinoids.^4–6^

A key feature of this novel approach is the incorporation of a polycyclic carbon cage into the porphyrin macrocycle. The cage subunit’s unique characteristics facilitate the construction of systems with an unconventional three-dimensional architecture, which in turn determines the electronic and molecular structures (including chirality) and the resulting functionality.^7–11^ The two classes of compounds demonstrated numerous intriguing yet distinct features. Porphyrin macrocycles are defined by their planar, rigid structures and delocalized π-electron systems. They also act as versatile ligands able to coordinate metal ions in multiple binding modes. On the other hand, cage compounds exhibit rigid molecular architectures, severe ring strain, and unusual conformations. A noteworthy aspect of the chemistry of polycyclic cages is the successful activation of inert C–H bonds within the adamantyl moiety, enabling the formation of stable nickel and palladium complexes.^12,13^

The synthesis of a new class of calix[4]­pyrrole analogues has recently been reported by Senge et al. (Figure 1).^14^ A rigid bicyclo[1.1.1]­pentane (BCP) fragment has been successfully embedded within the flexible macrocyclic framework of calix[4]­pyrrole. This approach afforded two structurally distinct hybrid macrocycles: tetrapyrrolic system **1** and doubly N-confused derivative **2**, both of which showcase the impact of embedding a highly strained carbon scaffold within a tetrapyrrolic macrocyclic architecture.

**Figure 1:**
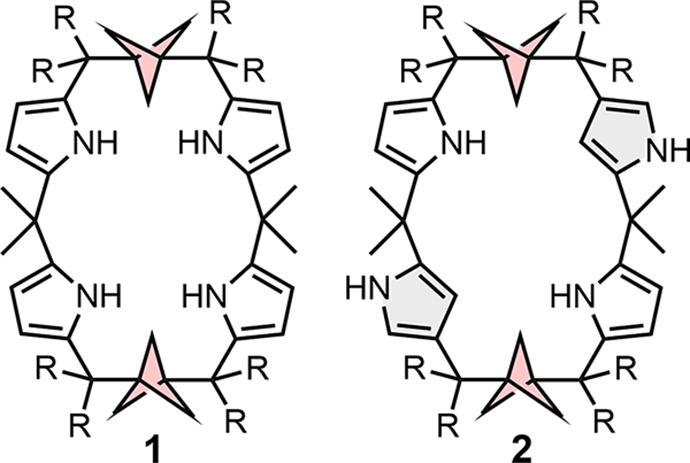
Calix­[4]­pyrrole­[2]­BCP derivatives **1** and **2** (one representative isomer is depicted, R = 4-FC_6_H_4_).

Here, we present a synthesis of another unique hybrid system, **3**, combining two distinct structural motifs: a planar porphyrin chromophore and a three-dimensional polycyclic carbon cage, bisnorditwistane, which comprises one cyclohexane and four cyclopentane rings (Figure 2).^15,16^ The design is conceptually grounded in the substitution of one pyrrolic unit within the parent heteroporphyrin framework by a rigid molecular cage, thereby generating a distinct class of carbaporphyrin architectures. From a structural and conceptual perspective, the macrocycle can be formally regarded as a derivative of tetrahydro-*p*-benziporphyrin.^17^

**Figure 2:**
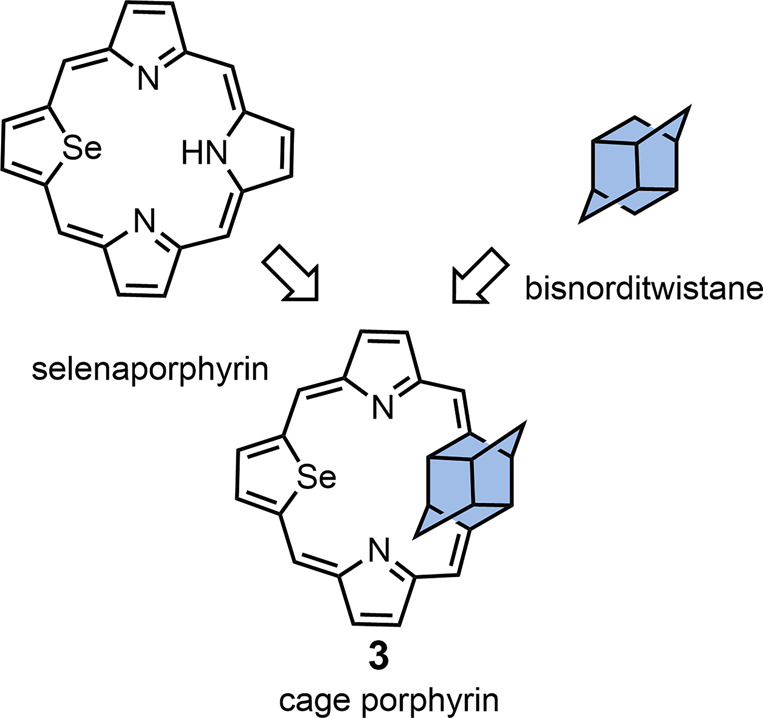
Fusion of selenaporphyrin and bisnorditwistane with the formation of a novel cage porphyrin **3**.

A key step in the synthesis of the porphyrin-cage molecule is the construction of suitable synthons for introducing the carbocyclic fragment ring into a porphyrin-like frame. We have used the synthetic method of Seo et al. to produce a mixture of isomeric 2-(cyclopentadienylmethyl)­pyrroles **4** (Scheme S1).^18^ These compounds undergo a [4+2] cycloaddition at 65 °C, affording a mixture of numerous cycloadducts **5** (Scheme S2), with the three major **5a**, **5c**, and **5e** being isolated in a ratio of 1.2:1:1.4 (Scheme 1). Unambiguous structural assignment of these products from the full set of possible isomers (Scheme S2) was achieved through detailed analysis of 2D NMR spectra. Utilization of compound **5a** as a synthetic synthon enabled the preparation of novel porphyrinoid framework **3** (Scheme 2).

**Scheme 1:**
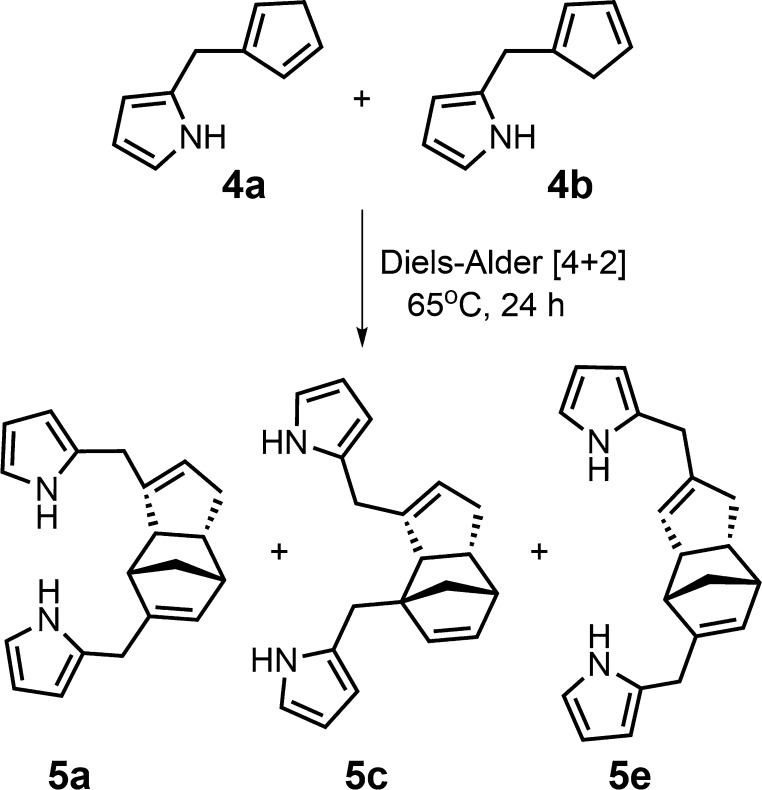
Synthesis of Cycloadducts 5

**Scheme 2:**
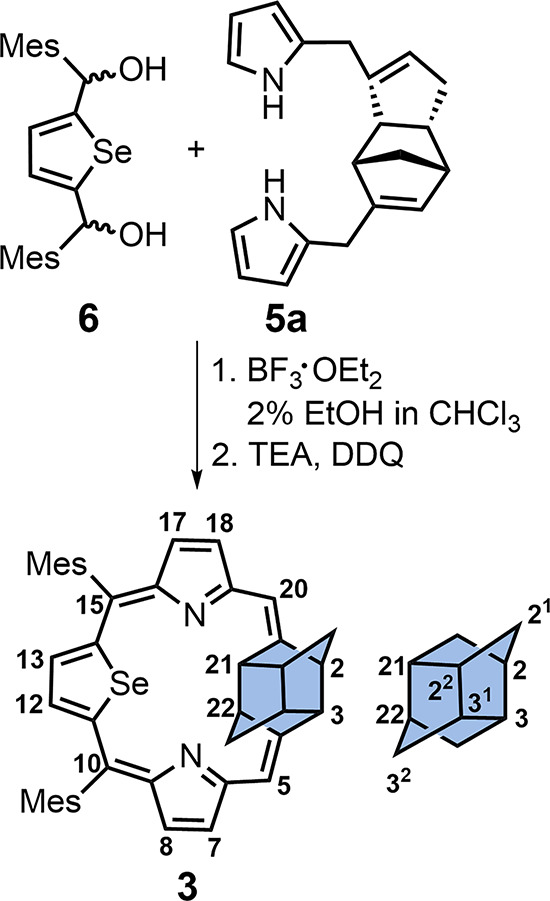
Synthesis of Cage Selenaporphyrin 3

The synthesis of selenaporphyrin-cage hybrid macrocycle **3** involves the one-pot reaction of 2,5-bis­(mesitylhydroxy-methyl)­selenophene **6** with **5a**, carried out in 2% (v/v) solution of ethanol in chloroform at 30 °C, catalyzed by BF_3_·OEt_2_, followed by oxidation with DDQ (2,3-dichloro-5,6-dicyano-1,4-benzoquinone) (Scheme 2). The procedure follows the [3+1] methodology previously utilized for the synthesis of carbaheteroporphyrinoids.^19–21^ Macrocycle **3** was isolated in 4.4% yield. The selection of the selenophene substrate **6** was inspired by recent studies on carbaselenaporphyrins and their unique reactivity.^21,22^

The tentative reaction mechanism posits that oxidation of the porphyrinogen **7** is accompanied by the formation of a new carbon–carbon bond “c” in the hypothetical intermediate **8**, leading to the bisnorditwistane structural motif (Scheme 3).

**Scheme 3:**
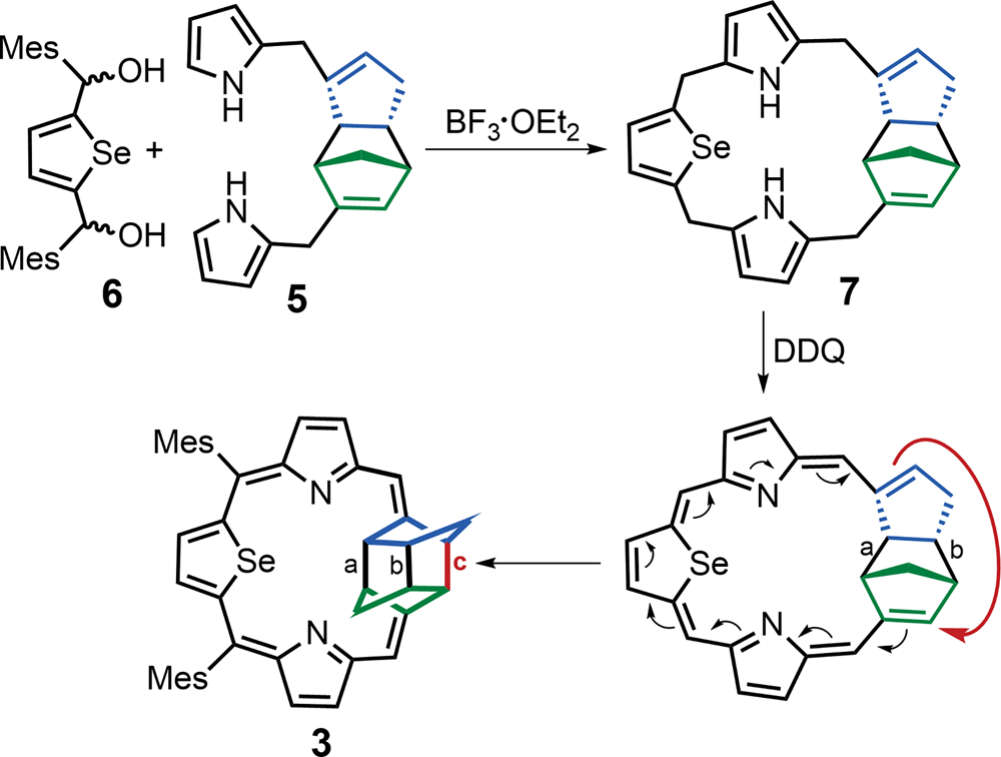
Proposed Mechanism of Bisnorditwistane Fragment Formation in 3

The resulting hybrid compound **3** is chiral and conformationally rigid. Single-crystal X-ray diffraction data indicate a reversible phase transition associated with twinning and a symmetry lowering on cooling (orthorhombic ↔ monoclinic) at about 220–240 K. Additionally, diffuse scattering was observed in the temperature range of 100–360 K (Figure S1). The average structure of the high-temperature phase is orthorhombic, described in the *P*2_1_2_1_2_1_ space group, with both enantiomers present in the unit cell; however, only one enantiomer of that kryptoracemic compound is depicted in Figure 3. Representations of both enantiomers, with full assignments of absolute configuration at all stereogenic centers, are provided in the Supporting Information (Figure S2). The macrocyclic fragment composed of two pyrrole units and a selenophene ring adopts an almost planar conformation, exhibiting only minor deviations of the heterocyclic rings from the plane defined by the four meso-carbon atoms. The polycyclic cage is placed outside this plane. A unique feature of the molecular structure of **3** is the short interatomic distance between selenium and the cage hydrogen H(22) (2.9 Å), which is shorter than the sum of the van der Waals radii.^23,24^ In the DFT-optimized structure of **3** this distance is equal to 2.8 Å.

**Figure 3:**
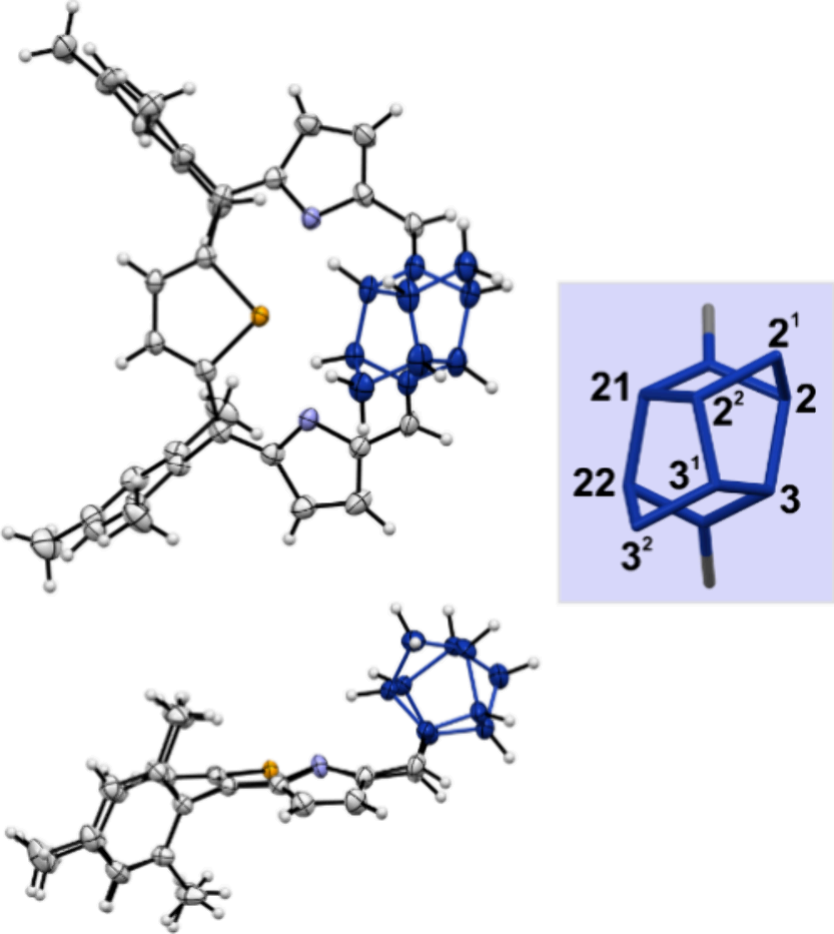
Molecular structure of compound **3** (one enantiomer is shown) displayed in two perspectives: a top view (top) and a side view (bottom). The bisnorditwistane fragment is illustrated on the right. The displacement ellipsoids represent 30% probability.

^1^H NMR spectrum of selenaporphyrin-bisnorditwistane compound **3** exhibits resonances in the region typical for nonaromatic porphyrinoids (Figure 4a). Protons of the π-conjugated fragment involving two pyrrole and one selenophene rings gave three AB spin systems within the 6.3–6.7 ppm region. The meso H(5) and H(20) protons appear as singlets at 6.06 and 5.73 ppm, respectively.

**Figure 4:**
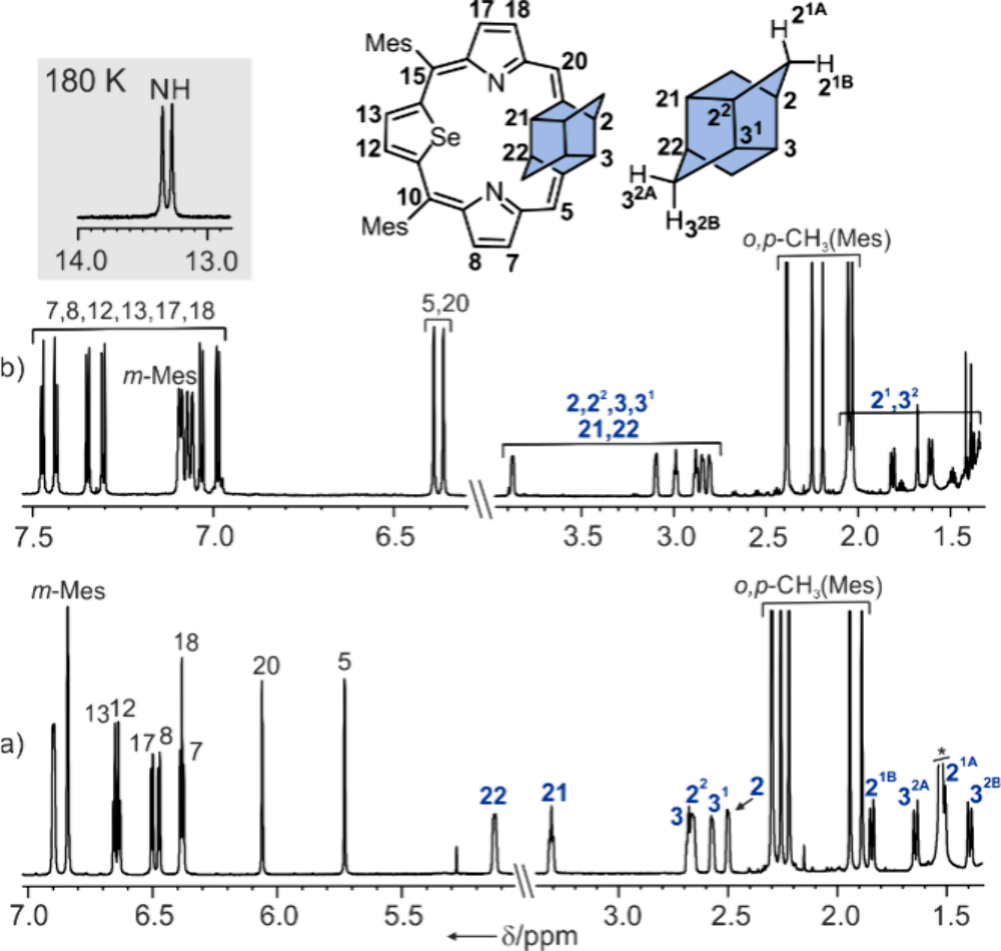
^1^H NMR spectra of a) **3** (300 K, CDCl_3_) and b) **3-H**_**2**_^**2+**^ (300 K, CD_2_Cl_2_).

The assignment of the rigid carbocyclic cage protons was made through careful analysis of the two-dimensional NMR spectra, particularly COSY, NOESY, and HSQC (Figures S27–S30). The crucial contacts observed in the NOESY spectrum are shown in Figure 5.

**Figure 5:**
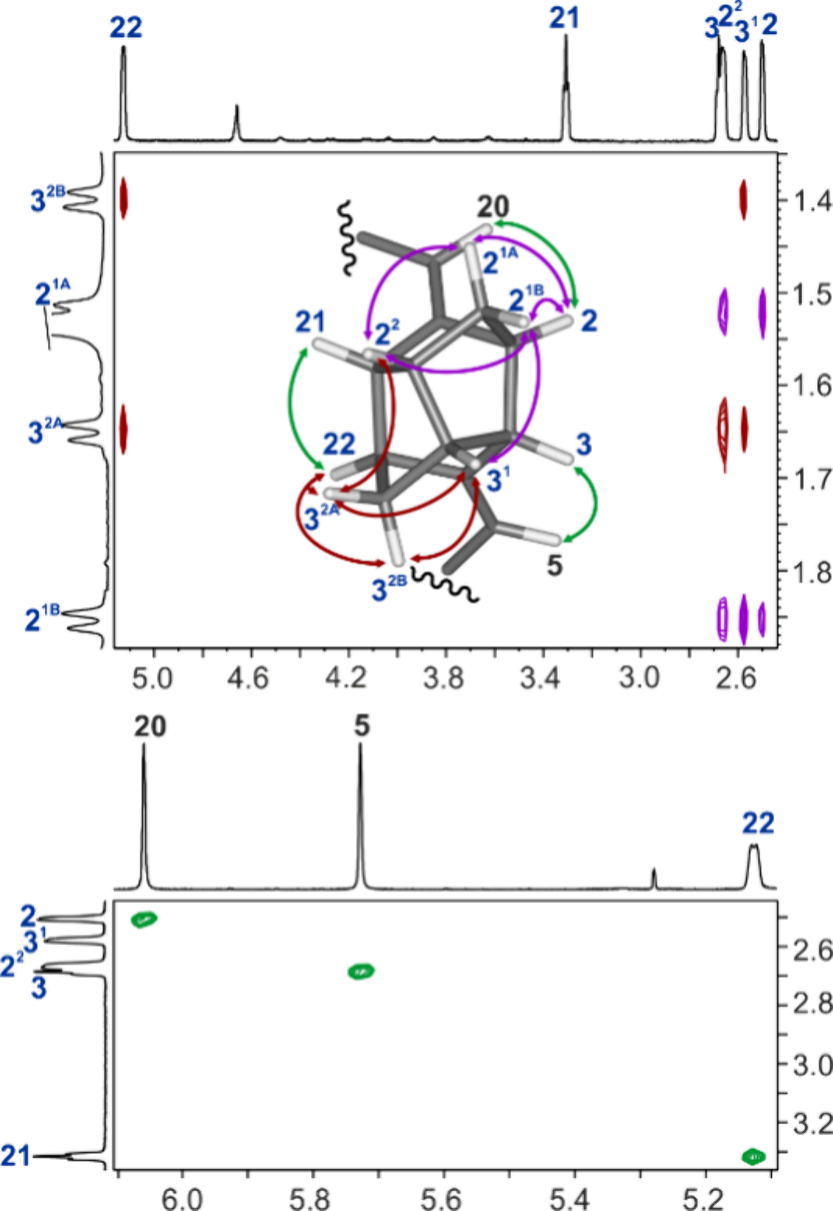
Crucial fragments of the ^1^H–^1^H NOESY spectrum of **3** (300 K, CDCl_3_).

Two CH_2_ and six CH protons of the cage fragment have been identified. The diastereotopically differentiated protons of two CH_2_ groups are localized in the 1.3–1.9 ppm range. External protons H(2) and H(3) of the cyclohexane ring of the cage gave signals at 2.50 and 2.65 ppm, respectively, while the inner protons H(21) and H(22) gave rise to resonances observed at 3.31 and 5.13 ppm.

The pronounced downfield shift of the H(22) resonance relative to the other cage-derived CH signals is attributed to an uncommon nonbonding C–H(22)···Se interaction, in agreement with previous reports.^23,24^ The presence of this rare hydrogen bond between the cage H(22) proton and the selenium atom is further corroborated by the ^1^H–^77^Se HMQC spectrum of **3** (Figure 6). The ^77^Se resonance is markedly downfield-shifted to 740 ppm relative to selenophene (605 ppm)^25^ and the ruthenium­(II) pentamethylcyclopentadienyl π-complex of carbaselenaporphyrin (598 ppm).^22^

**Figure 6:**
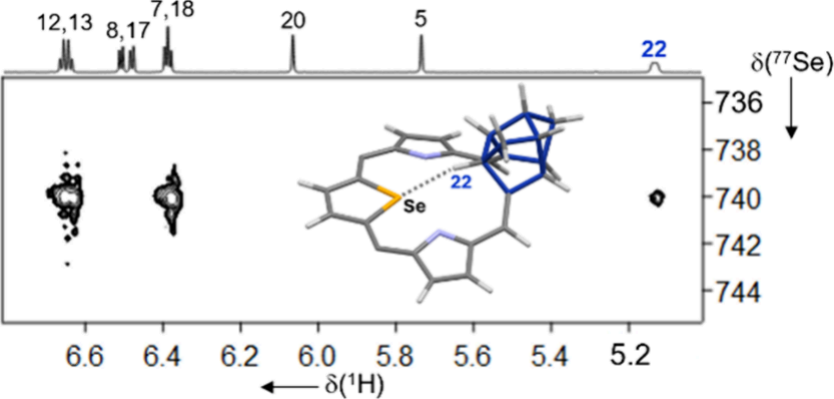
^1^H–^77^Se HMQC spectrum of **3** (300 K, CDCl_3_).

Furthermore, the ^13^C NMR spectrum revealed the presence of eight aliphatic carbon atoms within the cage (Figure S26). The analysis showed that two CH_2_ carbon atoms give signals at 36.8 [C­(3^2^)] and 36.2 ppm [C­(2^1^)], while six resonances of CH groups were observed at 50.8 [C(2)], 50.3 [C(22)], 49.1 [C­(2^2^)], 46.9 [C­(3^1^)], 46.8 [C(3)], 41.5 ppm [C(21)].

The experimental chemical shifts in both the proton and carbon spectra demonstrate a high degree of agreement with the corresponding calculated values from DFT studies (Table S2 and Figure S43).

A gradual acidification of compound **3** with trifluoroacetic acid (TFA) solution induces a distinct color transition from blue to green and, ultimately, yellow-brown, reflecting systematic changes in both the UV/vis absorption (Figures 7 and S38) and ^1^H NMR spectra (Figures 4b, S33, and S34). Protonation proceeds through a two-step sequence (Scheme 4).

**Figure 7:**
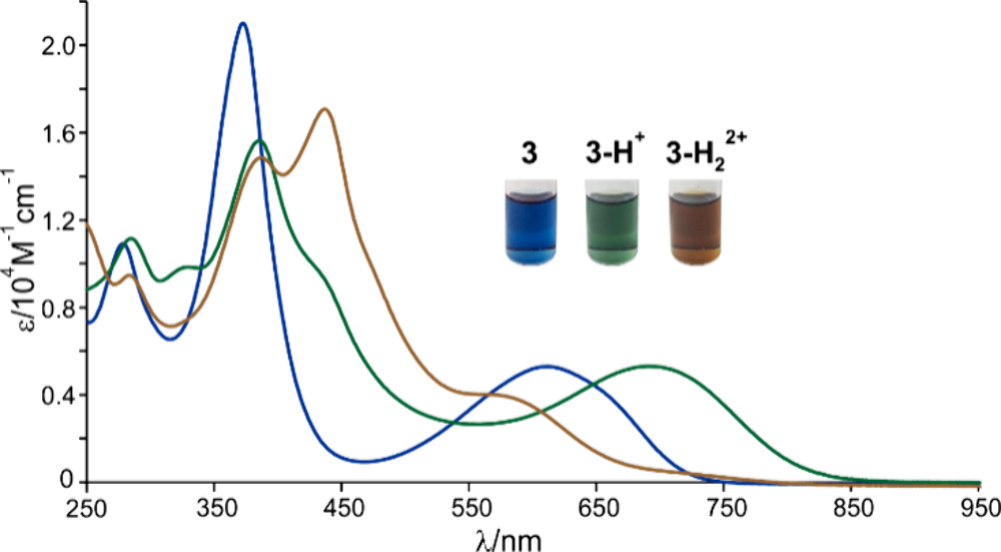
UV/vis spectra of **3** (blue), **3-H** ^**+**^ (green), and **3-H**_**2**_^**2+**^ (yellow-brown) in dichloromethane.

**Scheme 4:**
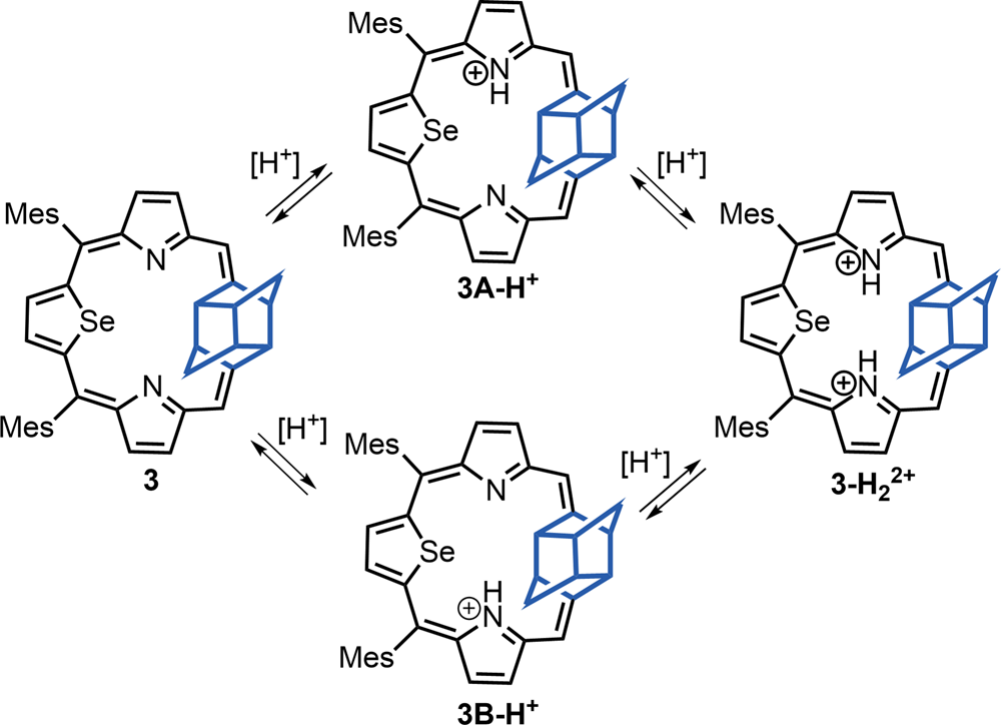
Protonation of **3**

In the resulting monocation, two tautomeric forms, **3A-H** ^**+**^ and **3BH** ^**+**^, are possible, differing in the position of the protonated pyrrolic nitrogen. The electronic absorption spectrum of neutral molecule **3** displays three bands at 278, 373, and 610 nm. Upon initial acid addition, the reaction mixture undergoes protonation to afford monocation **3-H** ^**+**^, characterized by absorption bands at 285, 330, and 385 nm, as well as an intense, markedly bathochromically shifted broad band centered at 691 nm. Progression of protonation leads to the formation of the final dication **3-H**_**2**_^**2+**^, evidenced by the appearance of absorption bands at 283, 387, and 437 nm, accompanied by a broad band at 565 nm exhibiting a pronounced hypsochromic shift relative to the neutral and monocationic species.

Furthermore, the monocationic and dicationic species were observed by ^1^H NMR during TFA titration at 180 K (Figures S33 and S34). During the controlled, stepwise addition of the acid solution, a gradual decrease in the signals of the neutral compound **3**, accompanied by the concurrent growth of the monocation **3-H** ^**+**^ and dication **3-H**_**2**_^**2+**^ resonances, was observed. For the monocation **3-H** ^**+**^, two NH resonances were observed (13.84 and 13.49 ppm at 180 K), consistent with the presence of two tautomeric forms (Scheme 3). A well-resolved spectrum of the fully protonated dication **3-H**_**2**_^**2+**^ was recorded at 300 K, whereas the NH proton signals are observable only at 180 K (Figures 4b and S33). In this spectrum, the protons of the triheterocyclic clamp in **3-H**_**2**_^**2+**^ appear markedly downfield, in the 7.5–6.9 ppm region, compared with the corresponding signals of the neutral compound **3**. The positions of the NH resonances exhibit a pronounced dependence on the acid concentration. At low TFA concentrations, the NH signals are observed at 15.43 and 15.14 ppm, whereas higher acid concentrations induce a substantial upfield shift to 13.35 and 13.27 ppm. The species observed during the titration, therefore, likely correspond to an initial dication interacting weakly with a single carboxylate anion, followed by the formation of more strongly bound complexes in the presence of excess TFA. At sufficiently high acid concentrations, the macrocycle is expected to incorporate one or more tightly associated carboxylate anions, resulting in distinct protonation–complexation equilibria reminiscent of those reported for other porphyrinoid systems.^26^

In summary, we have demonstrated a strategy for the three-dimensional functionalization of porphyrins that affords a new class of hybrid molecules integrating two fundamentally distinct structural elements. The resulting architecture combines a nearly planar π-conjugated triheterocyclic fragment with a rigid, chiral aliphatic carbocyclic cage – bisnorditwistane, producing an unprecedented spatial arrangement.

The macrocycle undergoes a well-defined two-step protonation sequence. The incorporation of carbon atoms from the three-dimensional cage carbon atoms into the macrocyclic core gives rise to a *C*_*n*_ *NXN*-type cavity that offers a promising coordination motif.

Furthermore, the availability of multiple cycloaddition products of 2-(cyclopentadienylmethyl)­pyrroles provides access to a broader family of hybrid macrocycles incorporating diverse carbocyclic motifs.

Importantly, the hybrid molecule is chiral and conformationally rigid, properties that may prove advantageous for probing the stereochemical aspects of molecular recognition, including interactions with anions, cations, and optically active acids. Given the unique three-dimensional arrangement and electronic characteristics of these systems, their potential application in asymmetric photocatalysis also merits consideration. These findings highlight the potential of carbon-cage-containing ligands in transition-metal coordination chemistry and related functional applications.

## Supplementary Material



## Data Availability

The data underlying this study are available in the published article, in the Supporting Information, and openly available in Zenodo at https://zenodo.org/records/19683562.

## Notes

Details, characterization data, additional schemes, NMR and MS spectra, and X-ray crystallographic analysis for **3** (PDF)
