# Supplementary material for: Three-Dimensional Functionalization of Macrocycles: Porphyrin and Polycyclic Carbon Cage Hybrid
Source: Org Lett. 2026 Jun 8;28(24):7747–52. doi: 10.1021/acs.orglett.6c01942 (PMC13309007; doi:10.1021/acs.orglett.6c01942)
Supplement: Supplementary file 1 [file ol6c01942_si_001.pdf]

Three-Dimensional Functionalization of Macrocycles: Porphyrin and Polycyclic Carbon Cage Hybrid

Katarzyna Stasiak, Katarzyna Ślepokura, Aleksandra Szumańska, Michał J. Białek, Lechosław Latos-Grażyński, and Anna Berlicka*

Abstract: A three-dimensional functionalization strategy for porphyrins affords a hybrid architecture integrating a porphyrinic structural motif with a rigid, chiral bisnorditwistane cage. The resulting system combines a sterically demanding carbon framework with a planar triheterocyclic fragment. The macrocycle exhibits a well-defined two-step protonation behavior.

Table of Contents

1.	General Information	3
2.	Experimental Procedures	5
2.1.	Solvents and reagents	5
2.2.	Synthesis	5
3.	Figures and schemes	7
4.	Molecular structure of **3**	8
5.	NMR spectra	10
6.	UV/Vis spectra	37
7.	MS spectra	38
8.	DFT calculations	40
8.1.	Optimized structures	40
8.2.	Correlation between calculated and experimental NMR values	42
8.3.	Cartesian coordinates	43
9.	References	46
10.	Author Contributions	46

1. General information

NMR Spectroscopy. All ^{1}H and ^{13}C NMR spectra were recorded on high-field Bruker Advance III spectrometers (^{1}H frequency 600 and 500 MHz), equipped with broadband inverse or conventional gradient probe heads. Spectra were referenced to the residual solvent signals ($CDCl_3$, 7.24 ppm; CD_2Cl_2, 5.32 ppm). ^{13}C NMR spectra were recorded with ^{1}H broadband decoupling and referenced to solvent signals ($^{13}CDCl_3$, 77.0 ppm, $^{13}CD_2Cl_2$, 54.0 ppm). The ^{77}Se-^{1}H HMBC and ^{77}Se-^{1}H HMQC spectra were recorded on the JEOL JNM-ECZ500R 500 MHz spectrometer at room temperature and referenced to selenophene (δ = 605 ppm)[1,2] used as an internal standard.

Mass Spectrometry. High-resolution and accurate mass spectra were recorded using ESI (Electrospray) or APCI (Atmospheric Pressure Chemical) ionization technique on the Bruker qTOF compact.

UV/Vis Spectroscopy. Electronic spectra were recorded on a Varian Carry 60 UV-Vis spectrophotometer.

Theoretical calculations. Geometry optimization was carried out in unconstrained C1 symmetry in vacuo, with starting coordinates derived from a pre-optimized model using Gaussian software.[3] Harmonic frequencies were calculated using analytical second derivatives to verify local minimum achievement, and no negative frequencies were observed. The calculations were performed at B3LYP/6-31G(d,p) level of theory.[4,5] NMR shifts were calculated using the GIAO method, with TMS shieldings as the NMR reference.

X-ray data. X-ray quality crystals of **3** were prepared via a gradual evaporation of acetonitrile from a solution of **3**. Diffraction data collected at 100 K revealed that the crystals were twinned and exhibited monoclinic symmetry. Further variable-temperature (290 → 100 → 360 K) experiments indicated a reversible phase transition associated with twinning and a lowering of symmetry on cooling (orthorhombic ↔ monoclinic), at approximately 220-240 K. Additionally, diffuse scattering was observed on *hkl* layers with h = 2, 6, and 10, across the entire temperature range, i.e., 100-360 K. The diffuse scattering streaks appeared between the Bragg reflections, along the **c*** direction (Fig. S1). Finally, the diffraction data were collected at 270 K, and **the average structure of the high-temperature phase is presented here**. Diffuse scattering was not explicitly modeled, however it is responsible for the elevated R-factors reported and the deviations in molecular geometry.

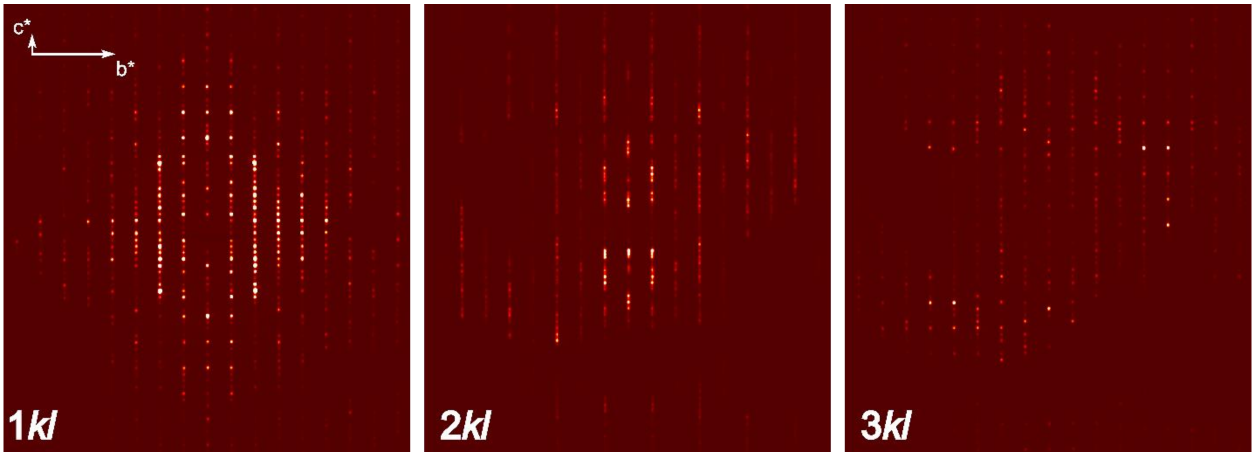

Figure S1. Ewald sphere reconstructions of the diffraction pattern of crystal **3**, with diffuse scattering visible in the 2*kl* layer. T = 270 K.

The measurement was performed on a κ-geometry four-circle diffractometer, Rigaku XtaLAB Synergy R, DW system (rotating anode X-ray source, ω scan method) using Cu Kα radiation and a hybrid HyPix-Arc 150° detector. Data were corrected for Lorentz and polarization effects and for absorption (using the empirical multi-scan method). Data collection, processing, and analysis were carried out with CrysAlis PRO.[6] The structure was solved using a dual-space algorithm implemented in the SHELXT program[7] and refined on F^2 by full-matrix least-squares technique using the SHELXL program,[8] with anisotropic displacement parameters applied to the ordered (fully occupied) and to the highly occupied positions of the disordered non-H atoms. For the structure solution and refinement, the Olex2 software was used.[9] The hydrogen atoms were included using geometrical considerations and refined using a riding model, with C–H = 0.93-0.98 Å, and with $U_{iso}(H) = 1.2U_{eg}(C)$ for CH and CH_2 or $1.5U_{eg}(C)$ for CH_3.

There are two molecules (denoted as A and B) in the asymmetric unit of crystal **3**. They were found to be disordered in two or even three positions each. Although it was possible to model two positions for molecules A and B (one of which always featured an occupancy of over 90%), in the final model, only the main positions of A and B (with the highest occupancy) were left. For the selenium atoms, three distinct positions were refined, with site-occupation-factors of 0.957(3), 0.036(2), and 0.0128(9) for Se1A, Se1X, and Se1Y, respectively, and 0.956(3), 0.038(2), and 0.0113(10) for Se1B, Se1Z, and Se1W. Restraints were applied to the sum of site-occupation-factors [1.000(2)] using the SUMP instructions in SHELXL. Geometrical restraints (SADI instructions in SHELXL) were applied to selected C–C bonds within the cage fragments of the two crystallographically independent molecules A and B. The crystal structure was refined as a two-component inversion twin (TWIN and BASF instructions).

The average structure of the high-temperature phase is orthorhombic and is described in the $P2_12_12_1$ Sohncke space group, with both enantiomers (denoted as A and B) present in the unit cell (Fig. S3). Therefore, **3** is a kryptoracemic compound. Molecules A and B form layers perpendicular to the **c**-axis (Fig. S4). Both within the layers and between them, C–H···π interactions exist. Considering the diffuse scattering, it might be assumed that the molecules are fully ordered within the layers (in the **ab** planes), whereas the crystal lacks periodicity (inter-layer correlation) in the third direction (along the **c**-axis), i.e., stacking faults are present and result in the one-dimensional (1D) diffuse effects along the **c***-axis. Since these diffuse streaks are present both below and above the temperature of phase transition, it can be concluded that the phase transition concerns some structural aspects within the layers, while the layer stacking faults remain unaffected. The disorder is likely not fully random; however, a comprehensive description of this phenomenon is beyond the scope of this paper.

The details of structure refinements are given in Table S1. The crystallographic information file (CIF) is deposited at the Cambridge Crystallographic Data Centre (CCDC No. 2549290) and provided as Supporting Information.

Table S1. Selected crystal data for **the average structure of the high-temperature phase of 3**.

CCDC	2549290
Chemical formula	$C_{44}H_{40}N_2Se$
M_r	676.13
Crystal system, space group	Orthorhombic, $P2_12_12_1$
Temperature (K)	270(2)
a, *b*, *c* (Å)	9.6224(16), 13.853(3), 51.631(8)
V (Å^3)	6882(2)
Z	8
Radiation type	Cu $K\alpha$
μ (mm^{-1})	1.73
Crystal size (mm)	0.16 × 0.08 × 0.01
Diffractometer	Rigaku, XtaLAB Synergy R, DW system with HyPix-Arc 150 detector
Absorption correction	Multi-scan
T_{min}, T_{max}	0.874, 1.000
No. of measured, independent and observed [$I > 2\sigma(I)$] reflections	69969, 12884, 11982
R_{int}	0.036
$(\sin \theta/\lambda)_{max}$ (Å^{-1})	0.615
$R[F^2 > 2\sigma(F^2)]$, $wR(F^2)$, S	0.061, 0.164, 1.06
No. of reflections	12884
No. of parameters	880
No. of restraints	39
H-atom treatment	H-atom parameters constrained
$\Delta\rho_{max}$, $\Delta\rho_{min}$ (e Å^{-3})	1.47, −0.61
Absolute structure	Refined as a 2-component inversion twin
Absolute structure parameter	0.21(3)

Computer programs: *CrysAlis PRO* 1.171.44.116a (Rigaku OD, 2025), SHELXT-2014/5 (Sheldrick, 2015), *SHELXL2014*/7 (Sheldrick, 2015).

2. Experimental procedures

2.1. Solvents and reagents

If not indicated differently, all solvents (chloroform, dichloromethane, ethyl acetate, n-hexane, ethanol, and methanol) were used without purification. $CDCl_3$ was prepared immediately before use by passing it through a basic alumina column. Reagents not listed here were used as received.

2.2. Synthesis

Cycloadducts 5 of isomeric 2-(cyclopentadienylmethyl)pyrroles 4

The isomeric 2-(cyclopentadienylmethyl)pyrroles **4** were prepared in two steps according to the reported procedure (cyclopentadiene: 10.35 g (156 mmol); pyrrole-2-carbaldehyde: 5.96 g (63 mmol); pyrrolidine: 8.94 g (126 mmol)) and used after chromatographic workup.[10] The isomers **4a** and **4b** were not separated before the next step.
Isomeric 2-(cyclopentadienylmethyl)pyrroles **4** (5.3 g, 36.5 mmol, yellow oil) were heated without solvent at 65 °C in an oil bath overnight. Preliminary purification of product **5** was carried out on a silica gel column using DCM as the eluent. The final separation of cycloadducts **5** was performed on silica gel (24 g column) by flash chromatography using 8% ethyl acetate in hexane (several small-scale chromatography runs gave the best results). Compound **5c** was eluted as the second fraction, **5e** as the third, and **5a** as the fourth. The first fraction contained unreacted substrate. Yields: 11% (1.13 g, pale yellow solid) for **5a**, 9% (0.95 g, pale yellow solid) for **5c**, and 13% (1.37 g, pale yellow oil) for **5e**. Compounds **5a**, **5c**, and **5e** were identified as the major cycloadducts formed in the reactions (see Scheme S2 and the corresponding discussion). Their structural assignments were established from COSY and NOESY analyses and supported by DFT-calculated models (see Fig. S41). The other cycloadducts (Scheme S2), formed in much lower yields, were not isolated in pure form and not characterized.

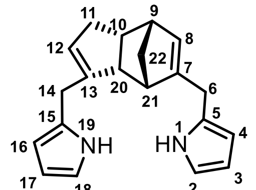

5a

5a: **^{1}H NMR** (600 MHz, $CDCl_3$, 300 K): δ = 7.91 (s, 1H, NH), 7.89 (s, 1H, NH), 6.66 (m, 2H, H2, H18), 6.12 (m, 2H, H(3),H(17)), 5.95 (m, 2H, H4, H(16)), 5.58 (m, 1H, H(8)), 5.21 (m, 1H, H(12)), 3.42 (m, 1H, H(14)), 3.33 (m, 2H, H(6), H(6’)), 3.27 (m, 1H, H(14’)), 3.05 (m, 1H, H(20)), 2.81 (m, 1H, H(9)), 2.78 (m, 1H, H(10)), 2.72 (m, 1H, H(21)), 2.17 (m, 1H, H(11)), 1.67 (m, 1H, H(11’)), 1.57 (m, 1H, H(22)), 1.27 ppm (m, 1H, H(22’)). Key diagnostic cross-peaks for stereoisomer **5a** identification were observed in the 2D NMR spectra: H(11)–H(12), H(11’)–H(12), H(8)–H(9) in the COSY spectrum, and H(8)–H(11′), H(11)–H(12), H(11’)–H(12) in the NOESY spectrum. **^{13}C NMR** (150.9 MHz, $CDCl_3$, 300 K): δ = 147.8, 143.6, 129.6, 129.4, 127.40, 127.37, 116.5, 108.3, 108.1, 106.12, 106.09, 56.0, 51.0, 47.8, 46.2, 42.5, 34.4, 30.0, 29.5 ppm. **HR-MS** (APCI): *m/z* calcd for $C_{20}H_{23}N_2^+$ $[M+H]^+$: 291.1856; found: 291.1860.

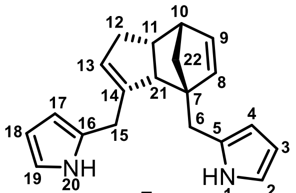

5c

5c: **^{1}H NMR** (600 MHz, $CDCl_3$, 300 K): δ = 8.04 (s, 1H, H(20)), 7.21 (s, 1H, H(1)), 6.80 (m, 1H, H(19)), 6.40 (m, 1H, H(2)), 6.27, (m, 1H, H(18)), 6.09 (m, 1H, H(17)), 6.06 (m, 1H, H(9)), 6.01 (m, 1H, H(3)), 5.87 (d, 1H, ^3J(H,H) = 5.7 Hz, H(8)), 5.75 (m, 1H, H(4)), 5.40 (m, 1H, H(13)), 3.59 (d, 1H, ^3J(H,H) = 16.2 Hz, H(15)), 3.44 (m, 1H, H(15’)), 3.03 (d, ^3J(H,H) = 14.9 Hz, 1H, (H(6)), 2.97 (d, ^3J(H,H) = 14.9 Hz, 1H, H(6’)), 2.80 (m, 2H, H(11), H(21)), 2.74 (m, 1H, H(10)), 2.19 (m, 1H, H(12)), 1.69 (m, 1H, H(12’)), 1.34 (m, 1H, H(22)), 1.15 ppm (m, 1H, H(22’)). Key diagnostic cross-peaks for stereoisomer **5a** identification were observed in the 2D NMR spectra: H(8)–H(9), H(9)–H(10), H(12)–H(13), H(12’)–H(13) in the COSY spectrum, and H(8)–H(9), H(9)–H(12′), H(6’)–H(15′), H(12)–H(13), H(12’)–H(13) in the NOESY spectrum.**^{13}C NMR** (150.9 MHz, $CDCl_3$, 300 K): δ = 141.5, 138.5, 133.9, 131.1, 129.8, 129.1, 117.3, 116.0, 108.4, 107.6, 106.6, 106.2, 57.7, 55.4, 54.2, 45.6, 45.1, 33.7, 30.6, 29.8 ppm. **HR-MS** (APCI): *m/z* calcd for $C_{20}H_{23}N_2^+$ $[M+H]^+$: 291.1856; found: 291.1856.

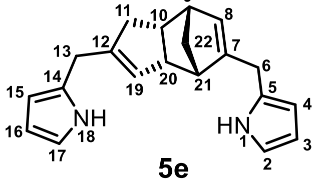

5e

5e: **^{1}H NMR** (600 MHz, $CDCl_3$, 300 K): δ = 7.86 (s, 1H, H(1)), 7.84 (s, 1H, H(18)), 6.67 (m, 1H, H(2)), 6.54 (m, 1H, h(17)), 6.14, (m, 1H, H(3)), 6.09 (m, 1H, H(16)), 5.95 (m, 1H, H(4)), 5.89 (m, 1H, H(15)), 5.47 (m, 1H, H(8)), 5.30 (m, 1H, H(19)), 3.4-3.2 (m, 5H, H(6), H(6’), H(13), H13’), H(20)), 2.77 (m, 2H, H(9), H(10)), 2.64 (m, 1H, H(21)), 2.07 (m, 1H, H(11)), 1.64 (m, 1H, H11’)), 1.57 (m, 1H, H(22)), 1.30 ppm (m, 1H, H22’)). Key diagnostic cross-peaks for stereoisomer **5e** identification were observed in the 2D NMR spectra: H(8)–H(9) in the COSY spectrum, and H(8)–H(11′) in the NOESY spectrum.

^{13}C NMR (150.9 MHz, $CDCl_3$, 300 K): δ = 148.8, 144.1, 129.5, 129.0, 127.4, 125.8, 116.7, 116.6, 108.01, 107.98, 106.1, 105.7, 54.0, 50.2, 49.0, 46.0, 41.9, 36.6, 30.4, 30.0 ppm. **HR-MS** (APCI): *m/z* calcd for $C_{20}H_{23}N_2^+$ $[M+H]^+$: 291.1856; found: 291.1846.

Selenaporphyrin-bisnorditwistane hybrid 3

120 mg (0.41 mmol) of **5a**, 210 mg (0.491 mmol) of **6**,[11] and a solution of 2% (v/v) EtOH in $CHCl_3$ (200 mL) were placed in a 500-mL flask. Nitrogen was bubbled through the solution for 20 min; then $BF_3{\cdot}OEt_2$ (112 µL, 0.91 mmol) was added, and the mixture was stirred for 1 h in the dark under nitrogen. Triethylamine (180 µL, 1.29 mmol) and DDQ (309 mg, 1.36 mmol) were added, and the solution was stirred for another 1 h under light. The solvent was removed, and the reaction mixture was initially purified by column chromatography on basic alumina (Brockmann III grade) using CH_2Cl_2 as the eluent. The residue was separated on a silica gel column (mesh 70-230). Impure **3** was eluted in the second blue fraction with CH_2Cl_2/*n*-hexane (1:4, V/V). After subsequent chromatography (silica gel, mesh 70-230; CH_2Cl_2/*n*-hexane (1:4, V/V)), 3 was obtained in the second fraction in 4.4% yield (12.2 mg). **UV/Vis:** λ_{max} ($\log\mathcal{E}$) = 278 (4.0), 373 (4.3), 610 nm (3.7).**^{1}H NMR** (600 MHz, $CDCl_3$, 300 K): δ = 6.90 (s, 1H, H(*m*-Mes), 6.89 (s, 1H, H(*m*-Mes), 6.84 (s, 2H, H(*m*-Mes)), 6.65 (d, ^3J(H,H) = 4.6 Hz, 1H, H(13)); 6.63 (d, ^3J(H,H) = 4.6 Hz, 1H, H(12)); 6.50 (d, ^3J(H,H) = 4.4 Hz, 1H, H(17)); 6.47 (d, ^3J(H,H) = 4.4 Hz, 1H, H(8)); 6.38 (d, ^3J(H,H) = 4.4 Hz, 1H, H(18)); 6.37 (d, ^3J(H,H) = 4.4 Hz, 1H, H(7)); 6.06 (s, 1H, H(20)); 5.73 (s, 1H, H(5)); 5.13 (m, 1H, H(22)); 3.31 (m, 1H, H(21)); 2.68 (m, 2H, H(3), H(2^2)); 2.57 (m, 1H, H(3^1)); 2.50 (m, 1H, H(2)); 2.30 (s, 6H, *p*-CH_3); 2.26 (s, 3H, 15-*o*-CH_3); 2.22 (s, 3H, 10-*o*-CH_3); 1.94 (s, 3H, 15-*o*-CH_3); 1.89 (s, 3H, 10-*o*-CH_3); 1.84 (m, 1H, H(2^{1B})); 1.64 (m, 1H, H(3^{2A})); 1.51 (m, 1H, H(2^{1A})); 1.39 ppm (m, 1H, H(3^{2B})).**^{13}C NMR** (150.9 MHz, $CDCl_3$, 300 K): δ = 168.3, 167.6, 162.9, 162.4, 161.5, 160.4, 154.5, 154.2, 142.9, 142.8, 137.19, 137.16, 137.14, 137.0, 136.6, 136.4, 134.3 (C(13)); 133.9 (C(12)), 133.7 (C(17)), 133.2, 133.1, 132.9 (C(8)), 129.4 (C(7)); 128.1 (C(18)), 127.9, 127.8, 127.7, 127.6, 113.6 (C(20)), 112.5 (C(5)), 50.8 (C(2)), 50.3 (C(22)), 49.1 (C(2^2)), 46.9 (C(3^1)), 46.8 (C(3)), 41.5 (C(21)), 36.8 (C(3^2)), 36.2 (C(2^1)), 21.0 (*p*-CH3), 20.4 (15-*o*-CH_3), 20.3 (10-*o*-CH_3), 20.1 (15-*o*-CH_3),19.8 ppm (10-*o*-CH_3). **^{77}Se NMR** ($CDCl_3$, 300 K, data from HMBC): δ = 740 ppm. **HR-MS** (ESI): *m/z* calcd for $C_{44}H_{41}N_2Se^+$ $[M+H]^+$: 677.2434; found: 677.2341.

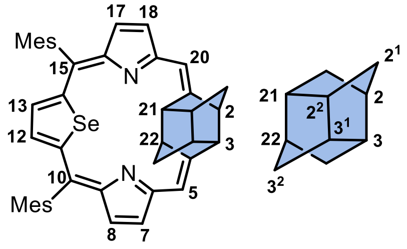

3

3-H$^+$ and **3-H$_2^{2+}$** were obtained by titrating **3** dissolved in CH_2Cl_2 or CD_2Cl_2 with TFA solution. The well-visible ^{1}H NMR spectrum was obtained only for **3-H$_2^{2+}$**.

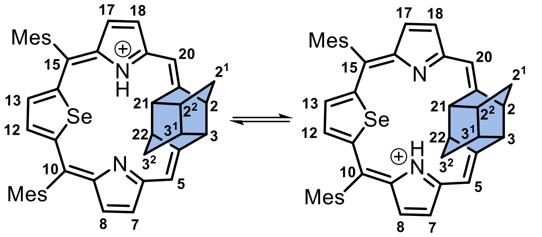

3-H$^+$

3-H$^+$: UV/Vis: λ_{max} ($\log\varepsilon$) = 285 (4.1), 330 (4.0), 385 (4.2), 691 nm (3.7).

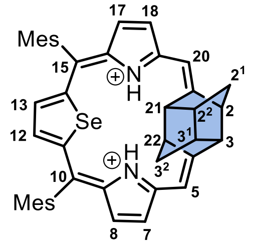

3-H$_2^{2+}$

3-H$_2^{2+}$: UV/Vis: λ_{max} ($\log\varepsilon$) = 283 (4.0), 387 (4.2), 437 (4.2), 565 nm (3.6).

^{1}H NMR (600 MHz, CD_2Cl_2, 180 K): δ 13.35 (s, 1H, NH), 13.27 (s, 1H, NH); 7.43 (d, 1H, pyrr/sel); 7.38 (d, 1H, pyrr/sel), 7.37 (d, 1H, pyrr/sel); 7.31 (s, 1H, pyrr/sel); 7.06—6.95 (m, 6H, H(m-Mes), pyrr/sel); 6.31 (s, 2H, H(5), H(20)); 3.70 (m, 1H, CH(cage)), 3.11 (m, 1H, CH(cage)), 2.93 (m, 1H, CH(cage)); 2.81 (m, 1H, CH(cage)); 2.74 (m, 1H, CH(cage)); 2.70 (m, 1H, CH(cage)); 2.30 (s, 3H, *p*-CH_3); 2.29 (s, 3H, *p*-CH_3); 2.14 (s, 3H, *o*-CH_3); 2.08 (s, 3H, *o*-CH_3); 1.95 (s, 3H, *o*-CH_3); 1.94 (s, 3H, *o*-CH_3); 1.91 (m, 1H, CH_2(cage)); 1.72 (m, 1H, CH_2(cage)); 1.48 (m, 1H, CH_2(cage)); 1.27 ppm (m, 1H, CH_2(cage)). **^{1}H NMR** (600 MHz, CD_2Cl_2, 300 K): δ 13.62, 13.68 (2s, 2H, NH); 7.48 (d, (d, ^3J(H,H) = 4.3 Hz, 1H, pyrr/sel); 7.44 (d, ^3J(H,H) = 4.3 Hz, 1H, pyrr/sel); 7.35 (d, ^3J(H,H) = 4.9 Hz, 1H, pyrr/sel); 7.31 (d, ^3J(H,H) = 4.3 Hz, 1H, pyrr/sel); 7.10 (s, 1H, H(m-Mes); 7.09 (s, 1H, H(m-Mes); 7.07 (s, 1H, H(m-Mes); 7.06 (s, 1H, H(m-Mes); 7.04 (d, ^3J(H,H) = 4.9 Hz, 1H, pyrr/sel); 6.99 (d, ^3J(H,H) = 4.9 Hz, 1H, pyrr/sel); 6.39 (s, 1H, H(5)/H(20)); 6.36 (s, 1H, H(5)/H(20)); 3.86 (m, 1H, CH(cage)), 3.10 (m, 1H, CH(cage)), 2.99 (m, 1H, CH(cage)); 2.88 (m, 1H, CH(cage)); 2.84 (m, 1H, CH(cage));

2.80 (m, 1H, CH(cage)); 2.39 (s, 3H, *p*-CH_3); 2.38 (s, 3H, *p*-CH_3); 2.25 (s, 3H, *o*-CH_3); 2.19 (s, 3H, *o*-CH_3); 2.05 (s, 3H, *o*-CH_3); 2.03 (m, 4H, *o*-CH_3, CH_2(cage)); 1.81 (m, 1H, CH_2(cage)); 1.60 (m, 1H, CH_2(cage)); 1.38 ppm (m, 1H, CH_2(cage)). NH resonances are not observable at 300 K.

3. Figures and Schemes

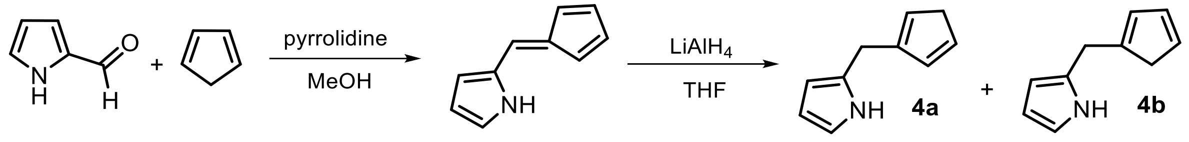

Scheme S1. Synthesis of **4**.[36]

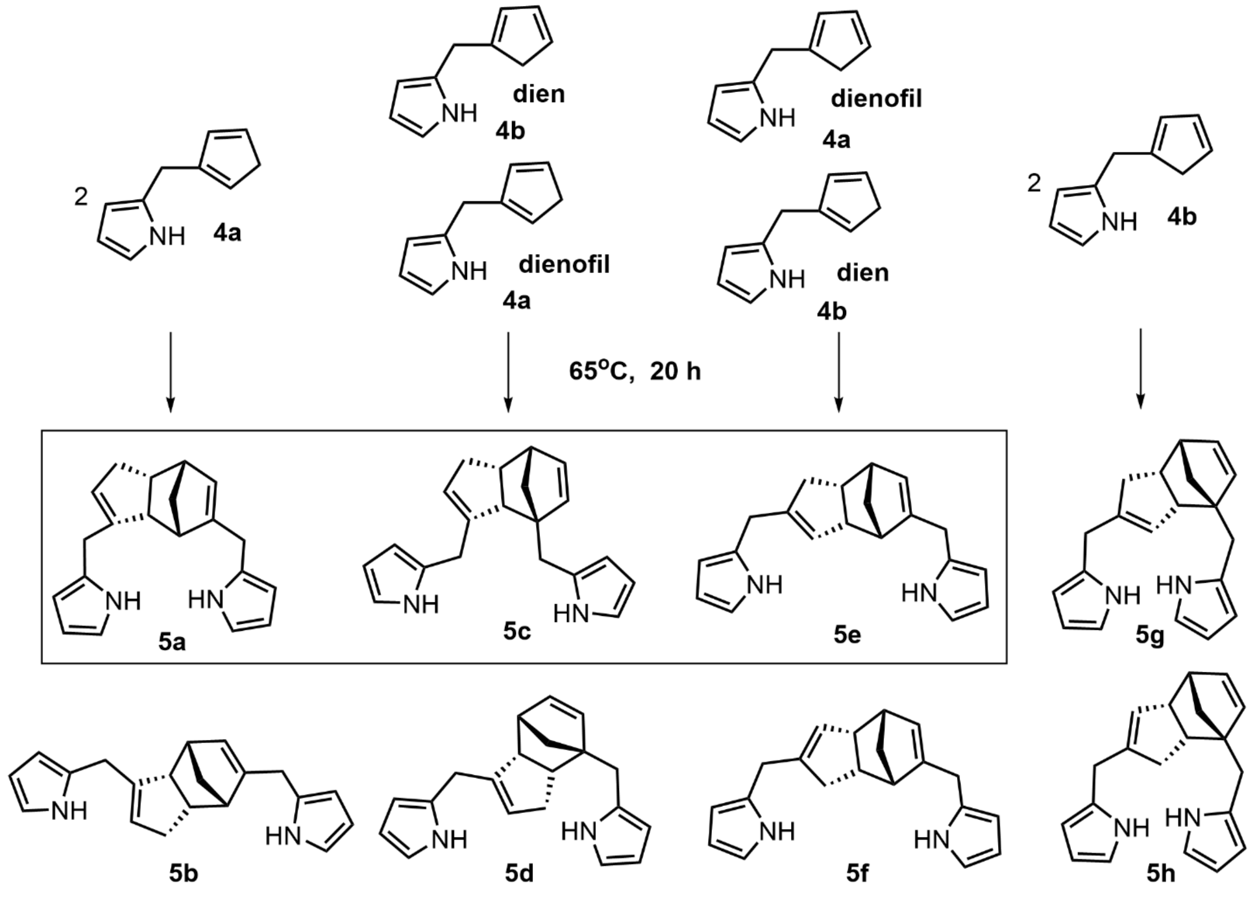

Scheme S2. Synthesis of **5**. All possible cycloadducts were initially taken into consideration, assuming that in each case the less substituted double bond of the cyclopentadiene unit serves as the dienophile and that the cycloaddition proceeds exclusively via the *endo* pathway. The remaining possible isomers were subsequently excluded based on a detailed analysis of the 2D NMR spectra (COSY and NOESY) and are therefore not presented here. Thorough examination of the 2D NMR data demonstrated that compounds **5a**, **5c**, and **5e** are the major products of the reaction. For clarity, only one enantiomer of each compound (**5a**-**5h**) is shown.

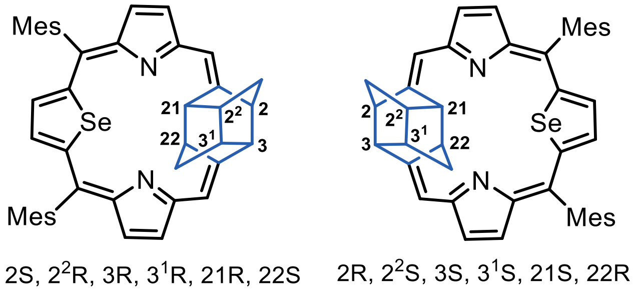

Figure S2. Two enantiomers of **3**.

4. Molecular structure of 3

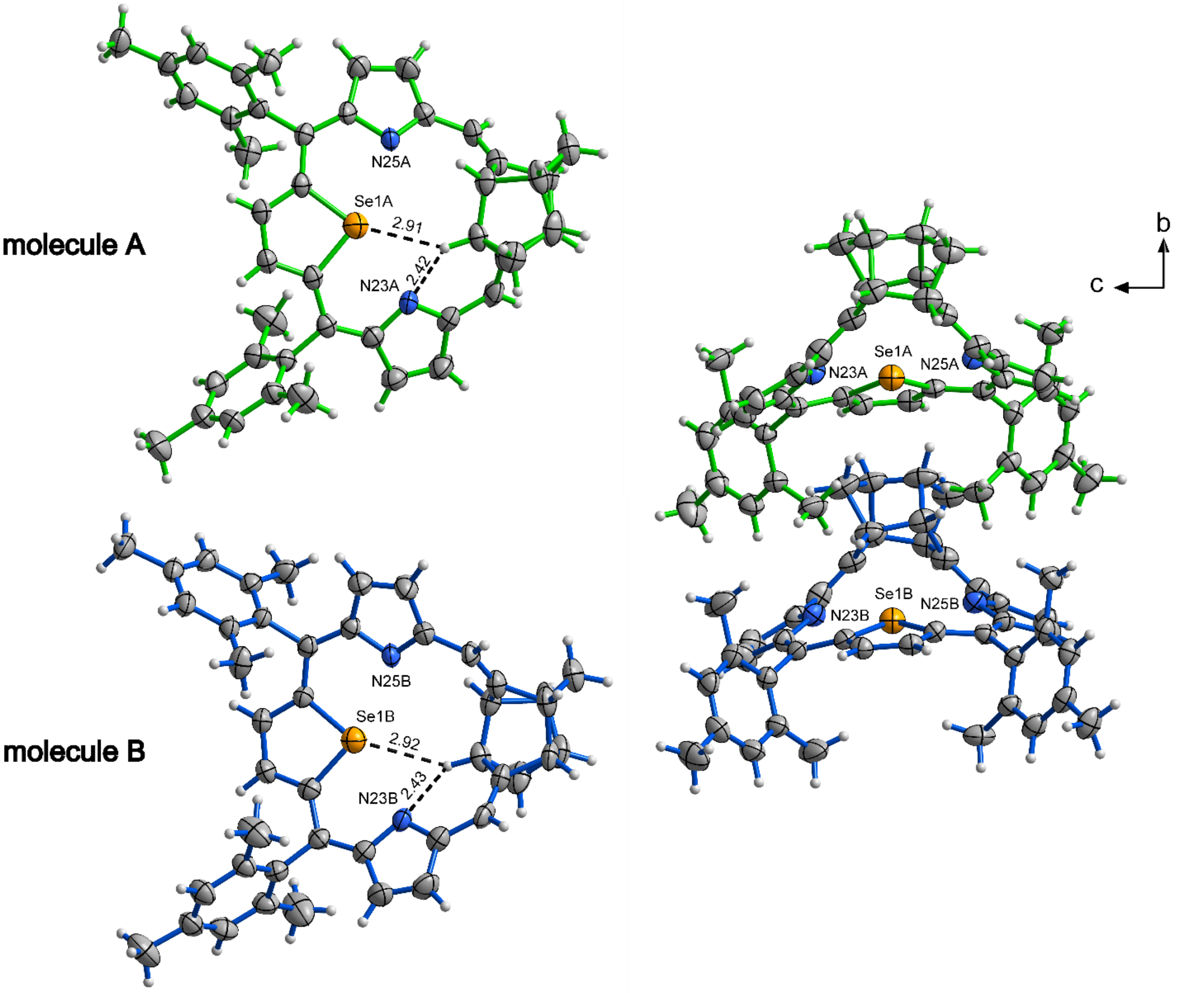

Figure S3. Crystallographically independent molecules A and B in crystal **3**, showing symmetry-independent C–H···Se/N hydrogen bonds (dashed lines, H···Se/N distances given in Å) (left), and their mutual orientation in the asymmetric unit (right). Displacement ellipsoids are shown at 50% probability level. The angle between two planes, one defined by four meso carbon atoms (C5, C10, C15, C20) and the other by two meso and two cage carbon atoms (C1, C20, C5, C4), is 66.0° in molecule A and 66.8° in molecule B.

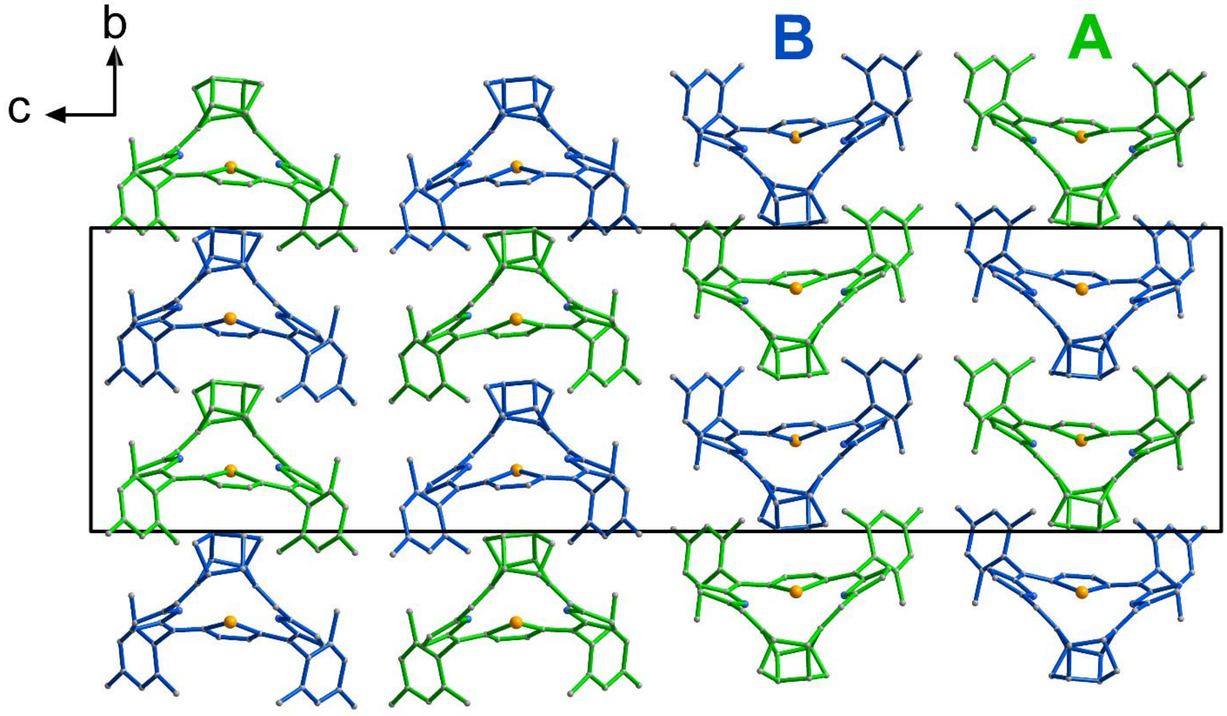

Figure S4. Packing of molecules in crystal **3**, viewed down the **a**-axis. H atoms are omitted for clarity.

5. NMR spectra

The NMR data are available at https://zenodo.org/records/19683562.

NMR of 5a

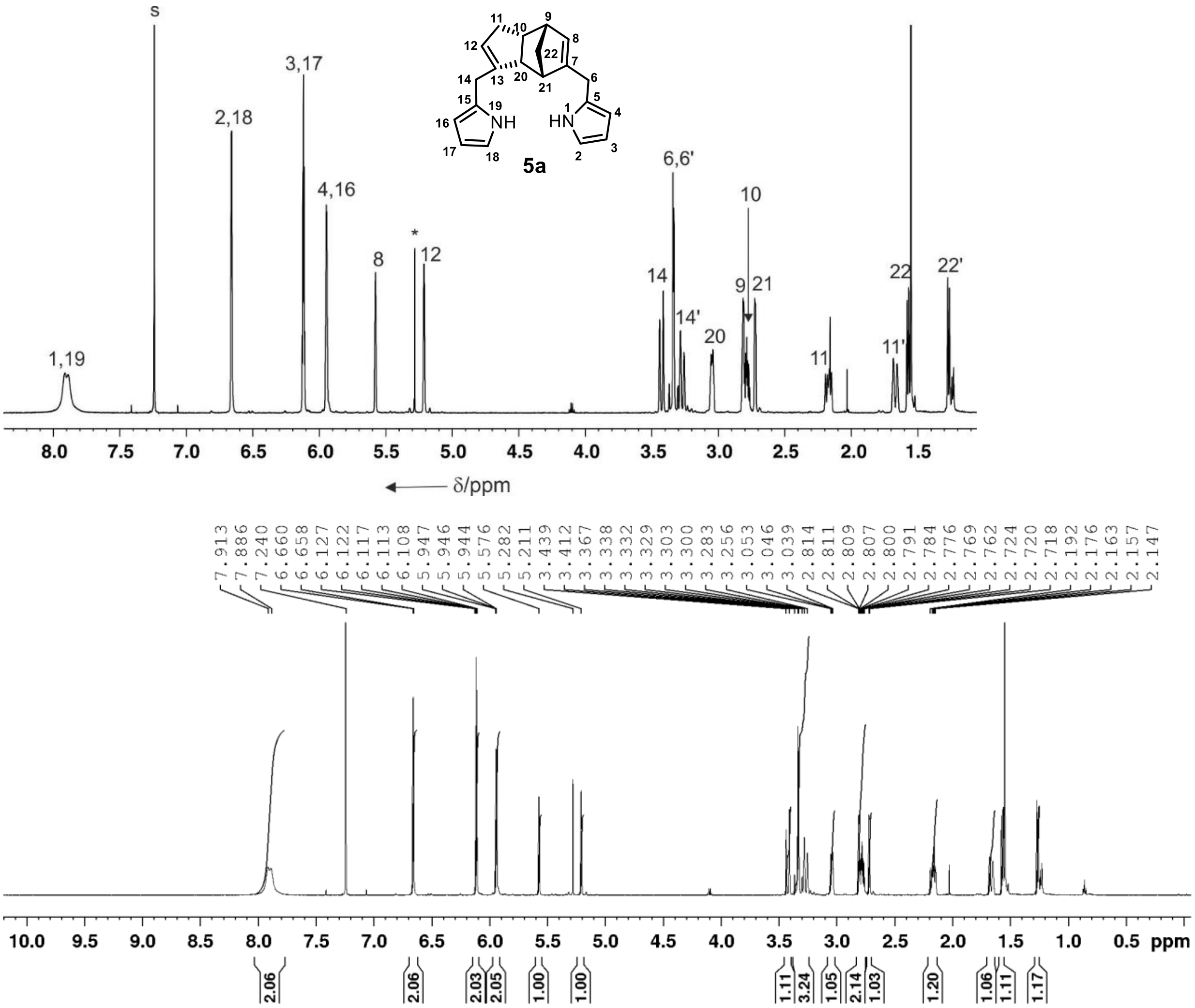

Figure S5. The ^{1}H NMR spectrum of **5a** ($CDCl_3$, 300 K); top: peak assignments.

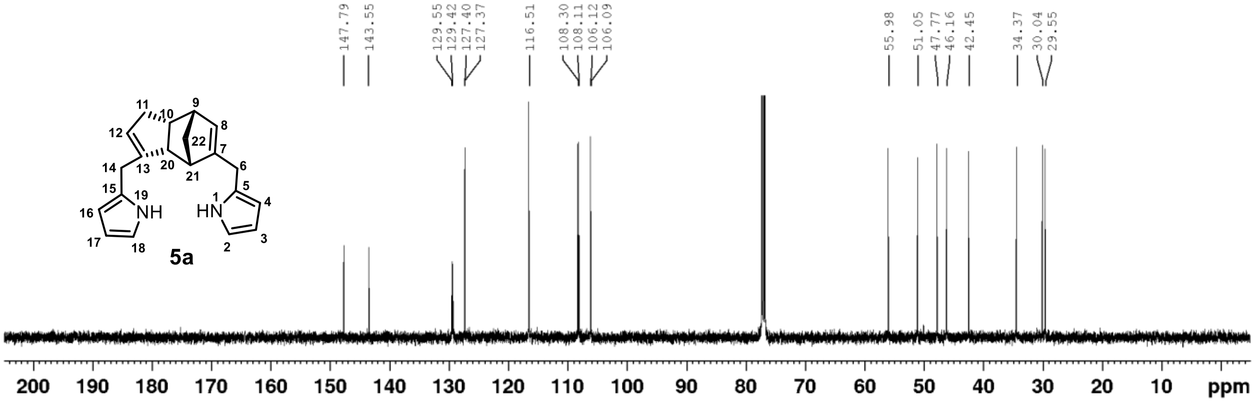

Figure S6. The ^{13}C NMR spectrum of **5a** ($CDCl_3$, 300 K).

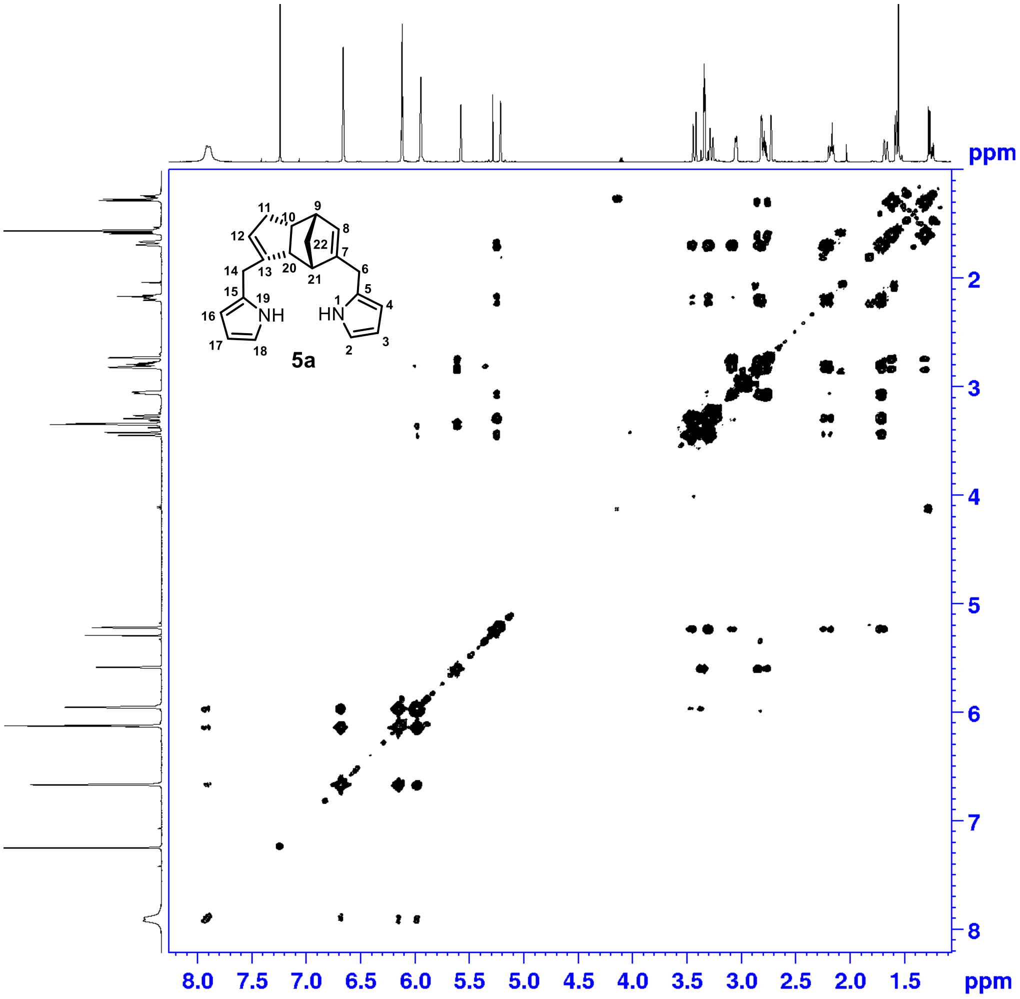

Figure S7. The ^{1}H-^{1}H COSY spectrum of **5a** ($CDCl_3$, 300 K).

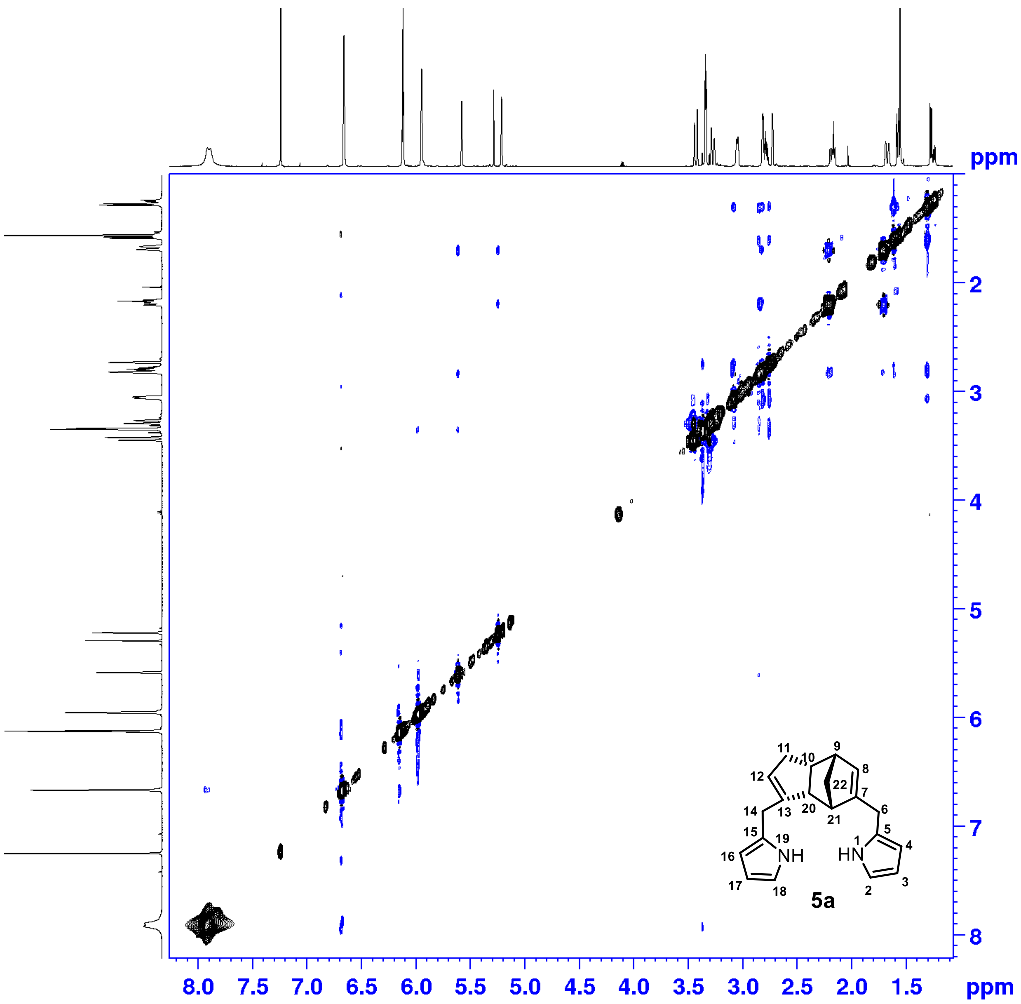

Figure S8. The ^{1}H-^{1}H NOESY spectrum of **5a** ($CDCl_3$, 300 K).

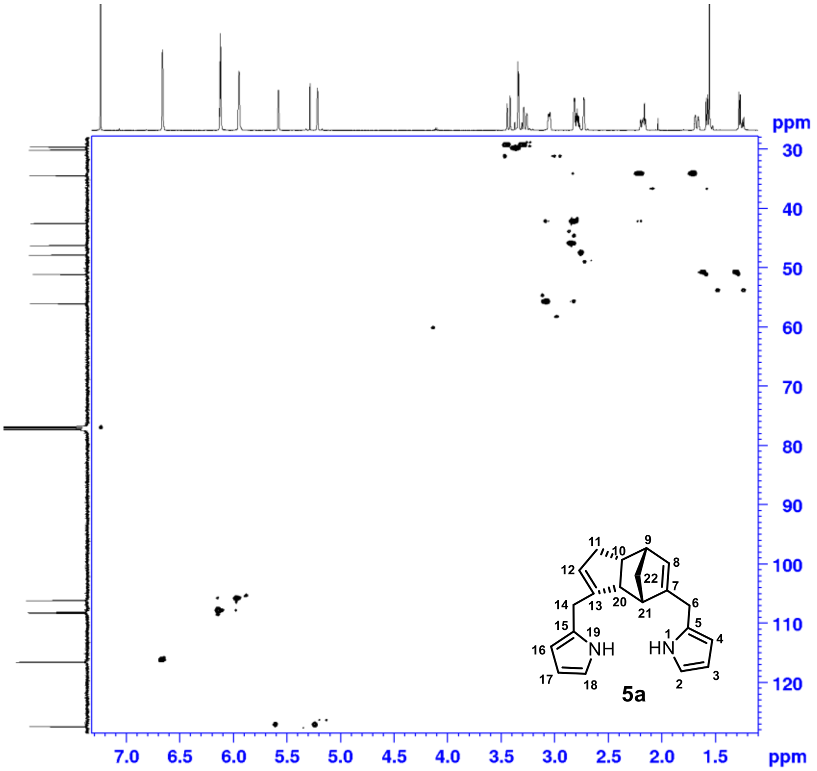

Figure S9. The ^{1}H-^{13}C HSQC spectrum of **5a** ($CDCl_3$, 300 K).

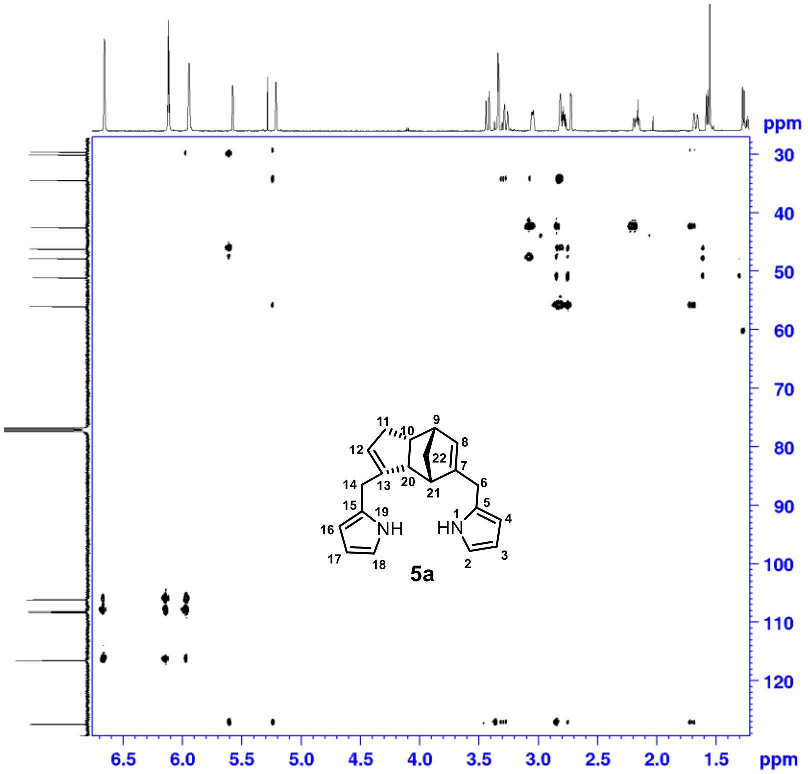

Figure S10. The ^{1}H-^{13}C H2BC spectrum of **5a** ($CDCl_3$, 300 K).

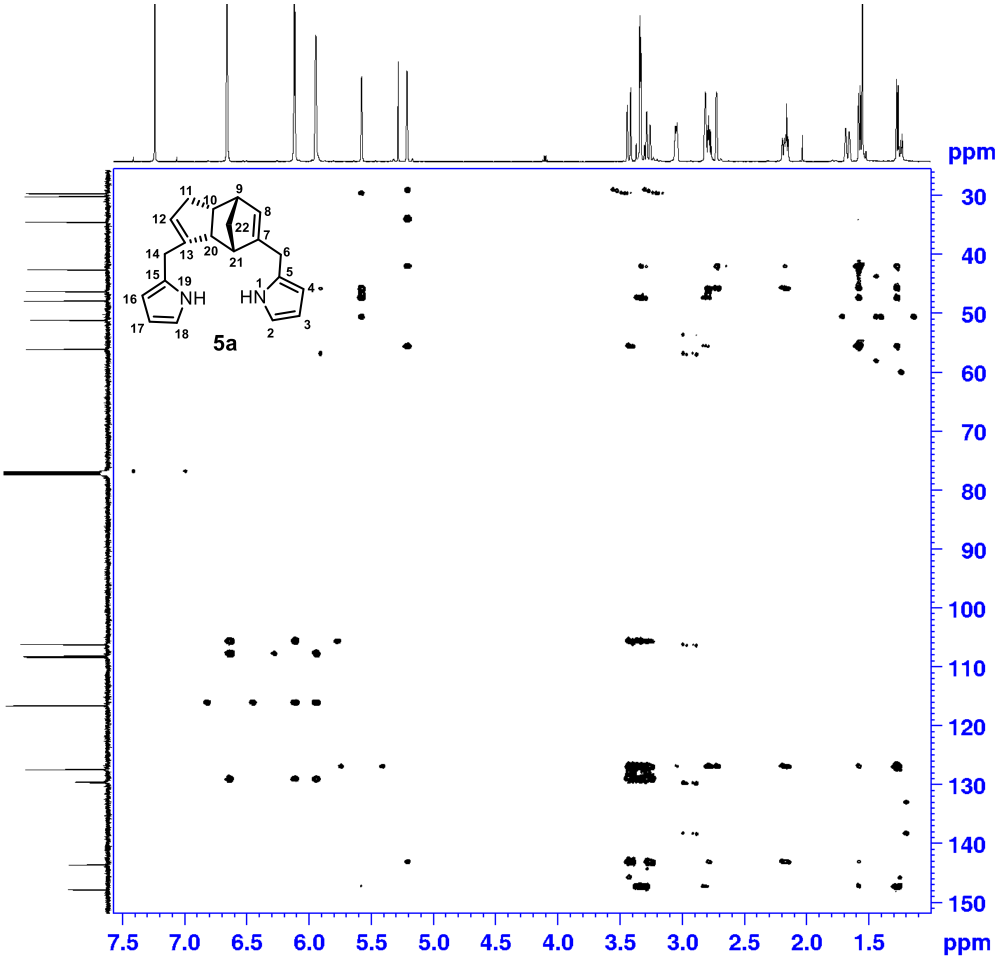

Figure S11. The ^1H-^{13}C HMBC spectrum of **5a** ($CDCl_3$, 300 K).

NMR of 5c

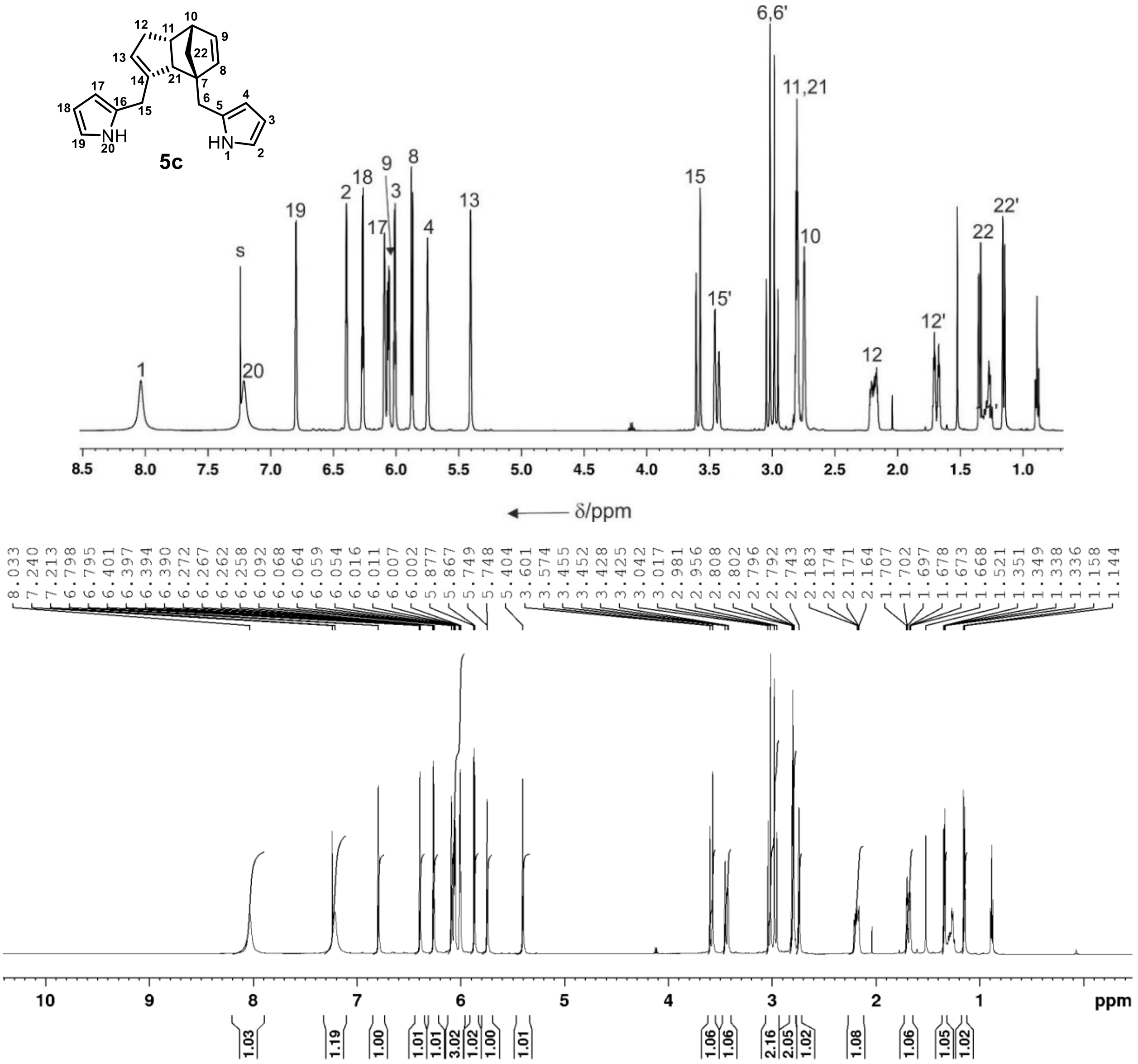

Figure S12. The ^{1}H NMR spectrum of **5c** ($CDCl_3$, 300 K); top: peak assignments.

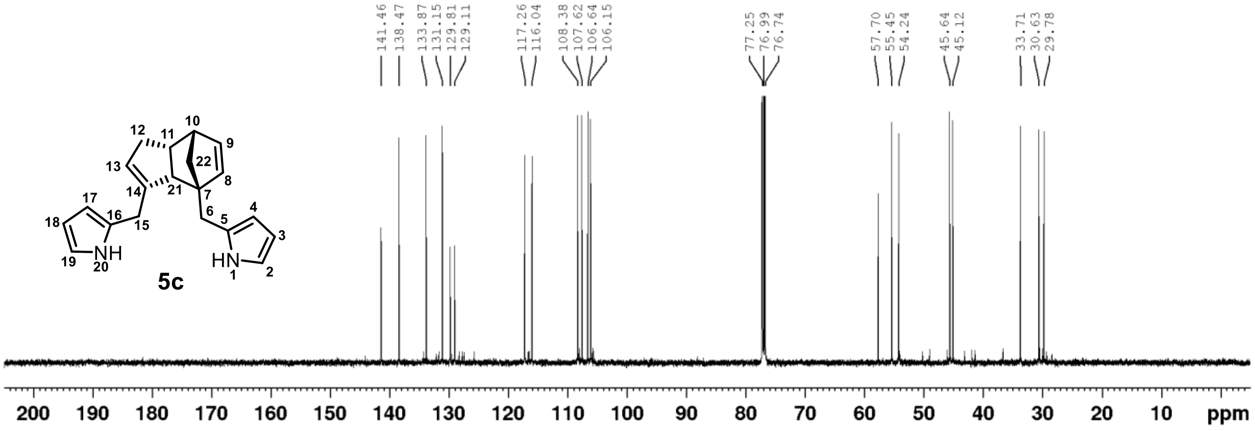

Figure S13. The ^{13}C NMR spectrum of **5c** ($CDCl_3$, 300 K).

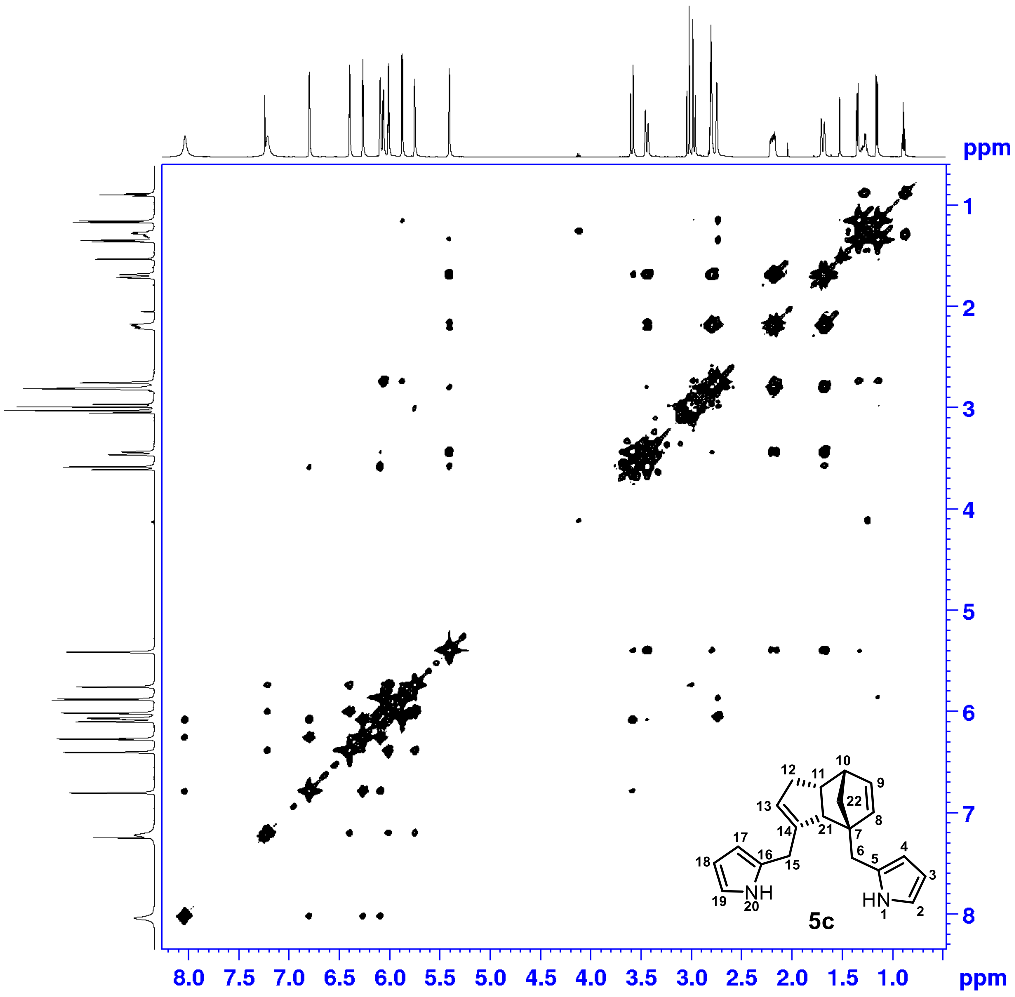

Figure S14. The ^{1}H-^{1}H COSY spectrum of **5c** ($CDCl_3$, 300 K).

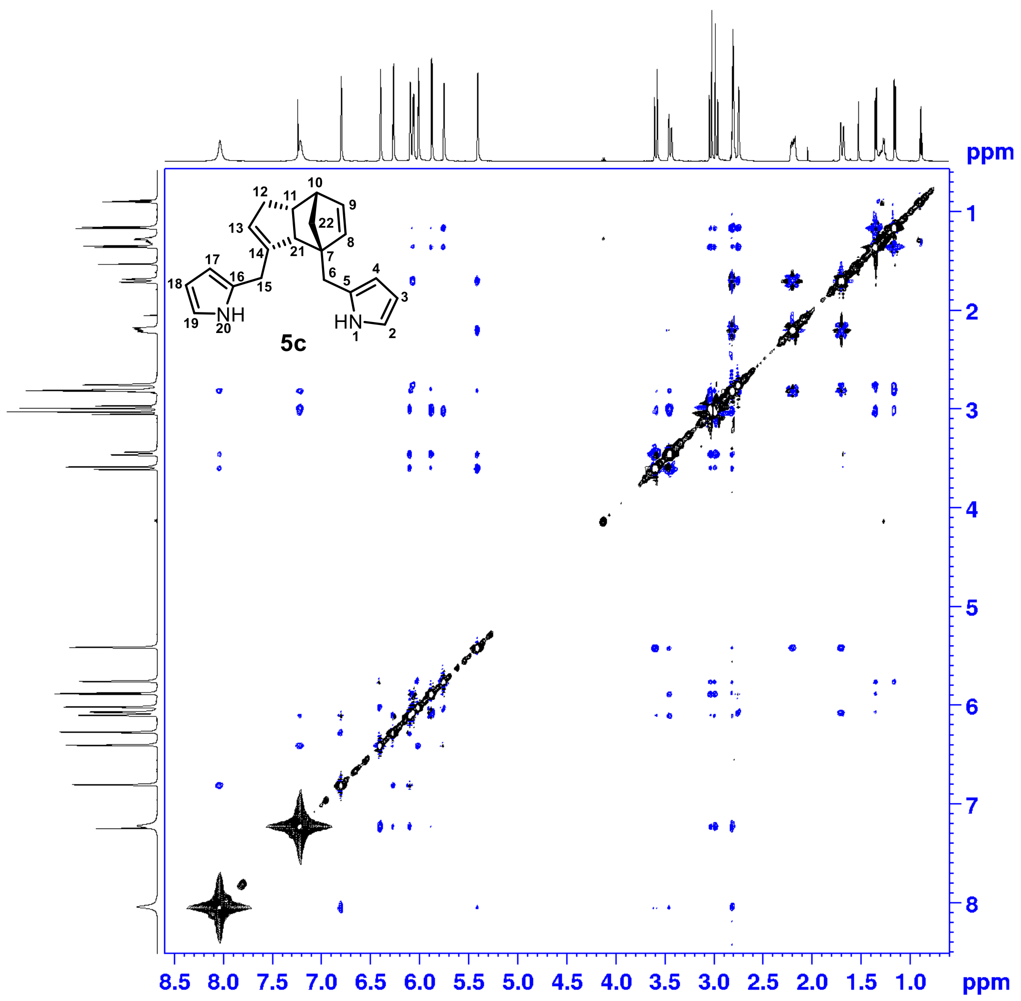

Figure S15. The ^{1}H-^{1}H NOESY spectrum of **5c** ($CDCl_3$, 300 K).

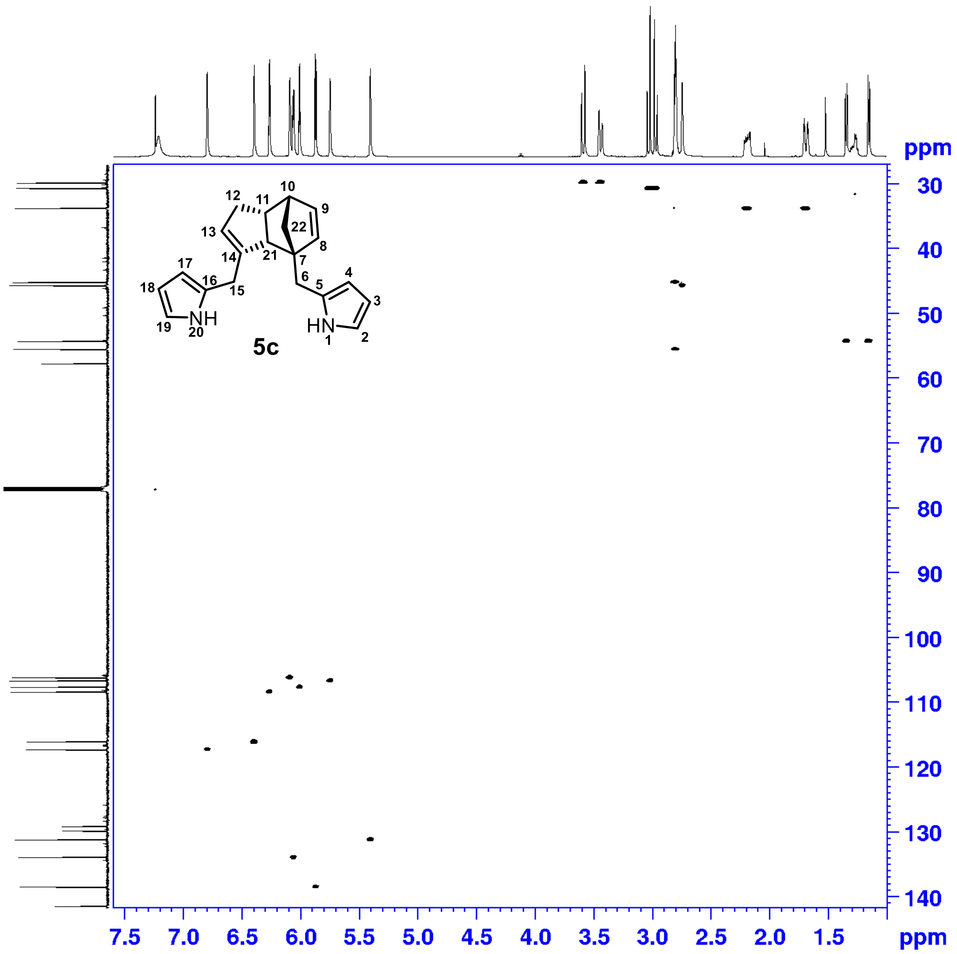

Figure S16. The ^{1}H-^{13}C HSQC spectrum of **5c** ($CDCl_3$, 300 K).

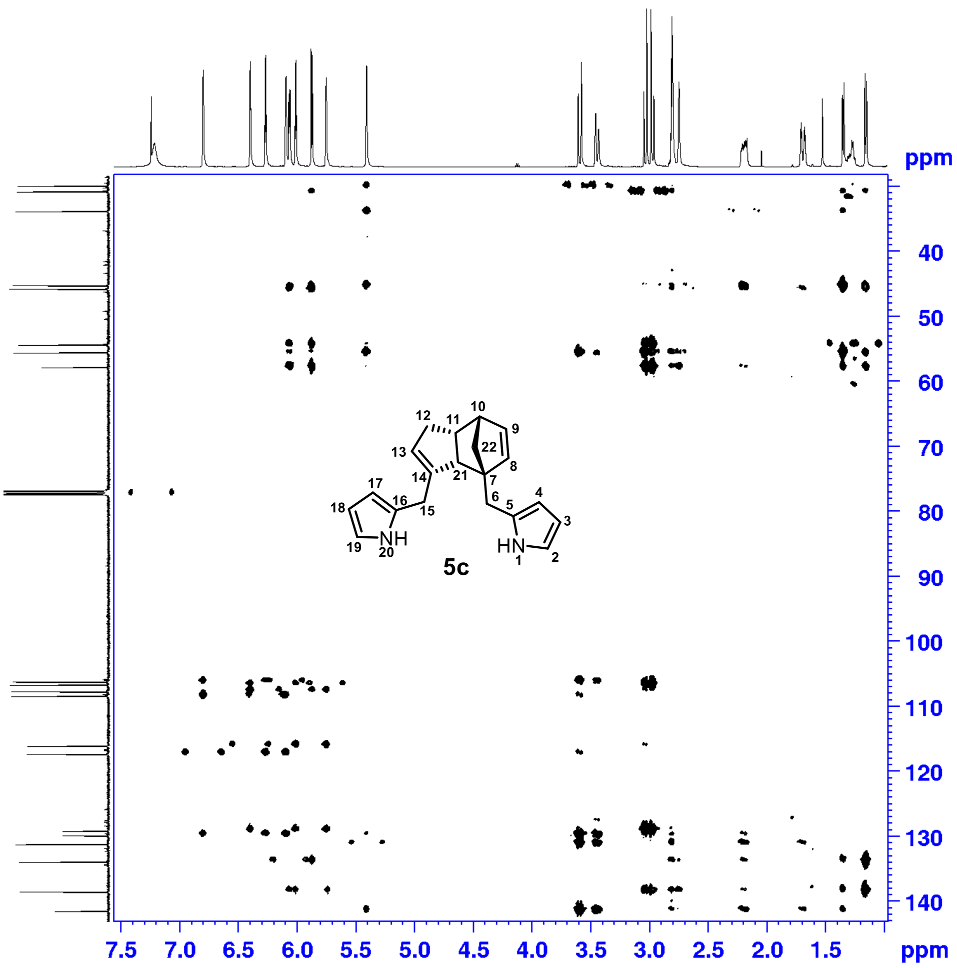

Figure S17. The ^{1}H-^{13}C HMBC spectrum of **5c** ($CDCl_3$, 300 K).

NMR of 5e

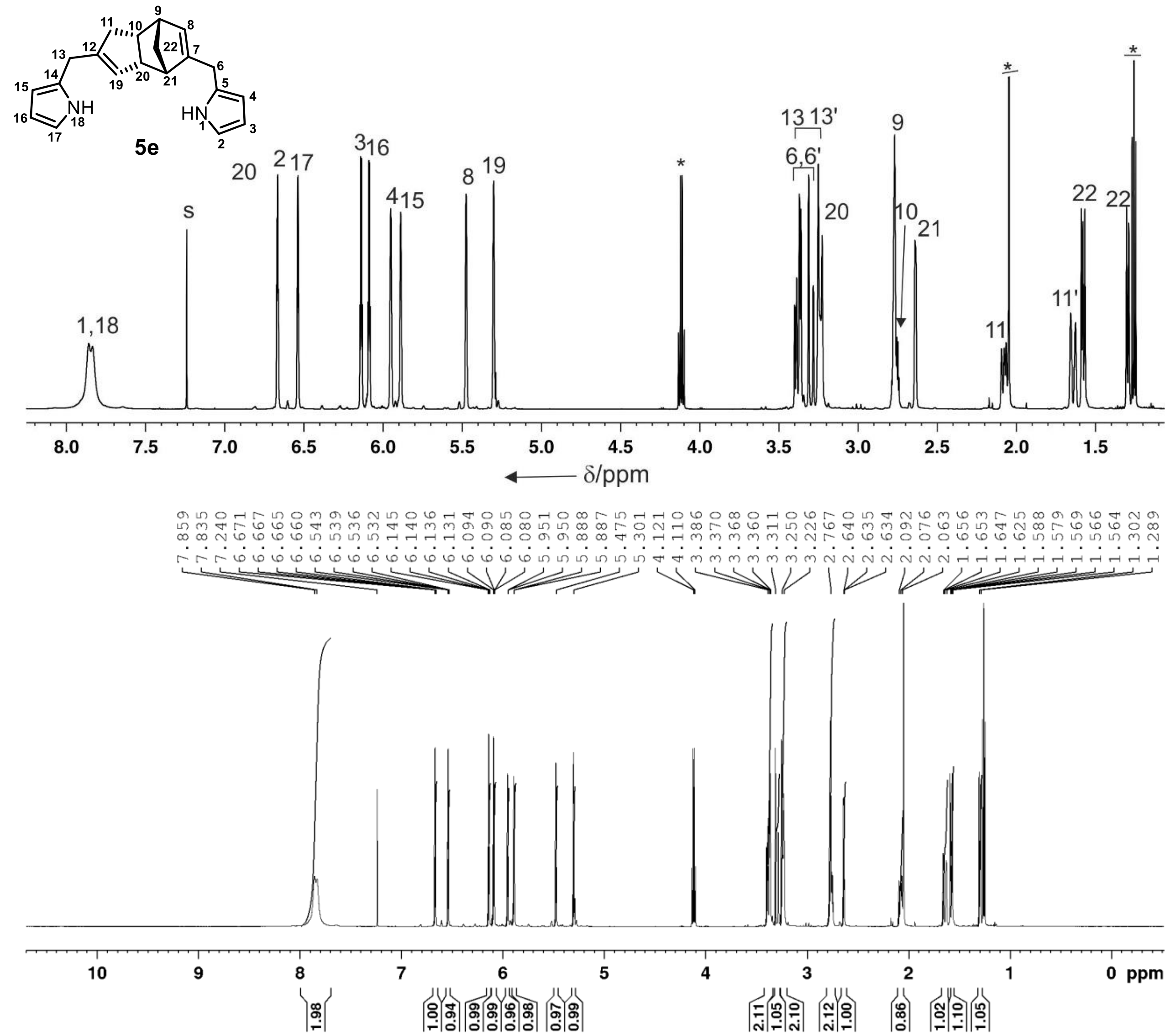

Figure S18. The ^{1}H NMR spectrum of **5e** ($CDCl_3$, 300 K), top: peak assignments.

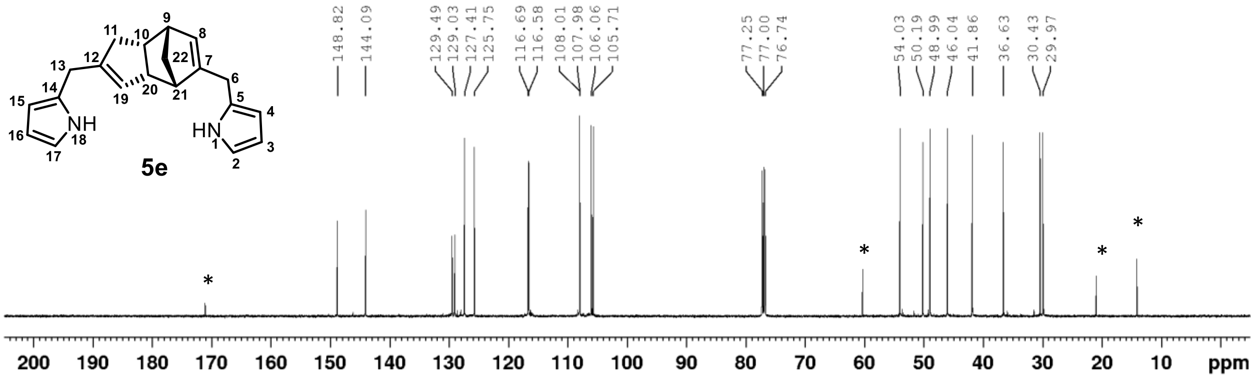

Figure S19. The ^{13}C NMR spectrum of **5e** ($CDCl_3$, 300 K).

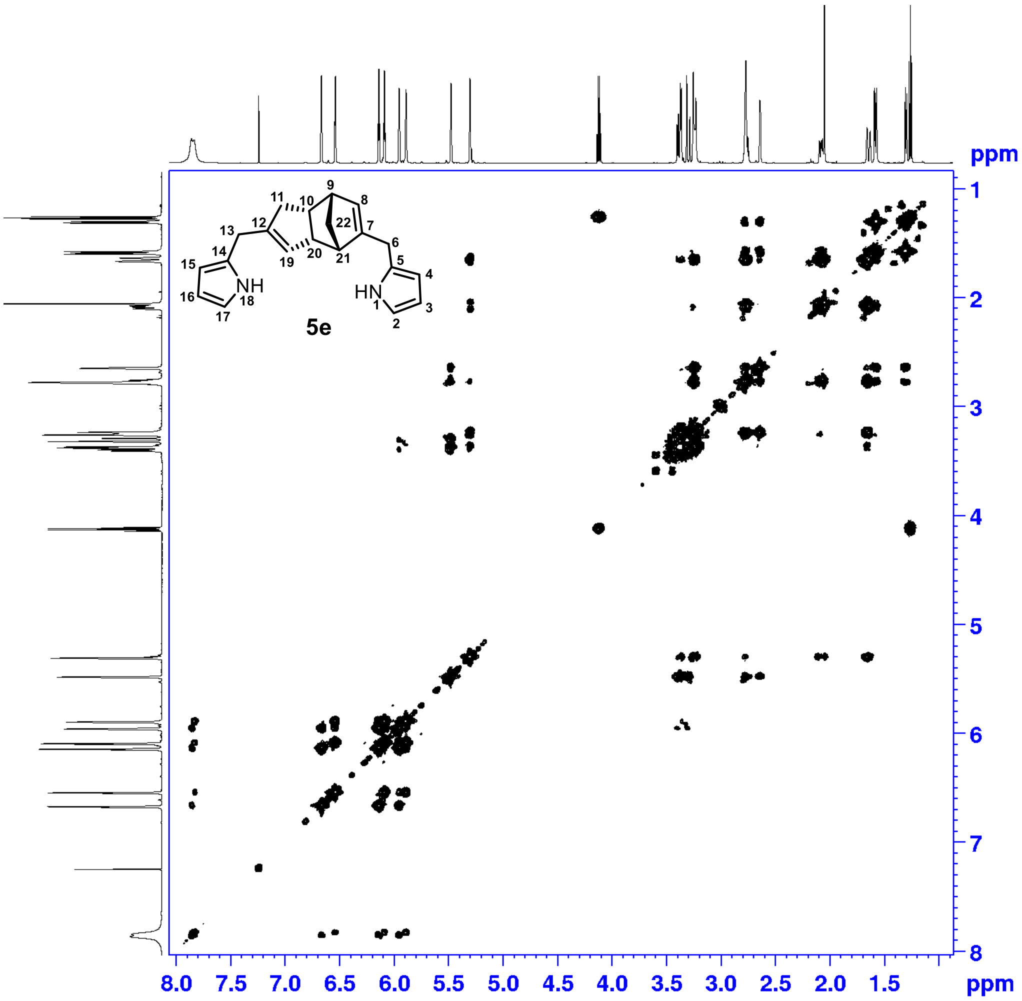

Figure S20. The ^{1}H-^{1}H COSY NMR spectrum of **5e** ($CDCl_3$, 300 K).

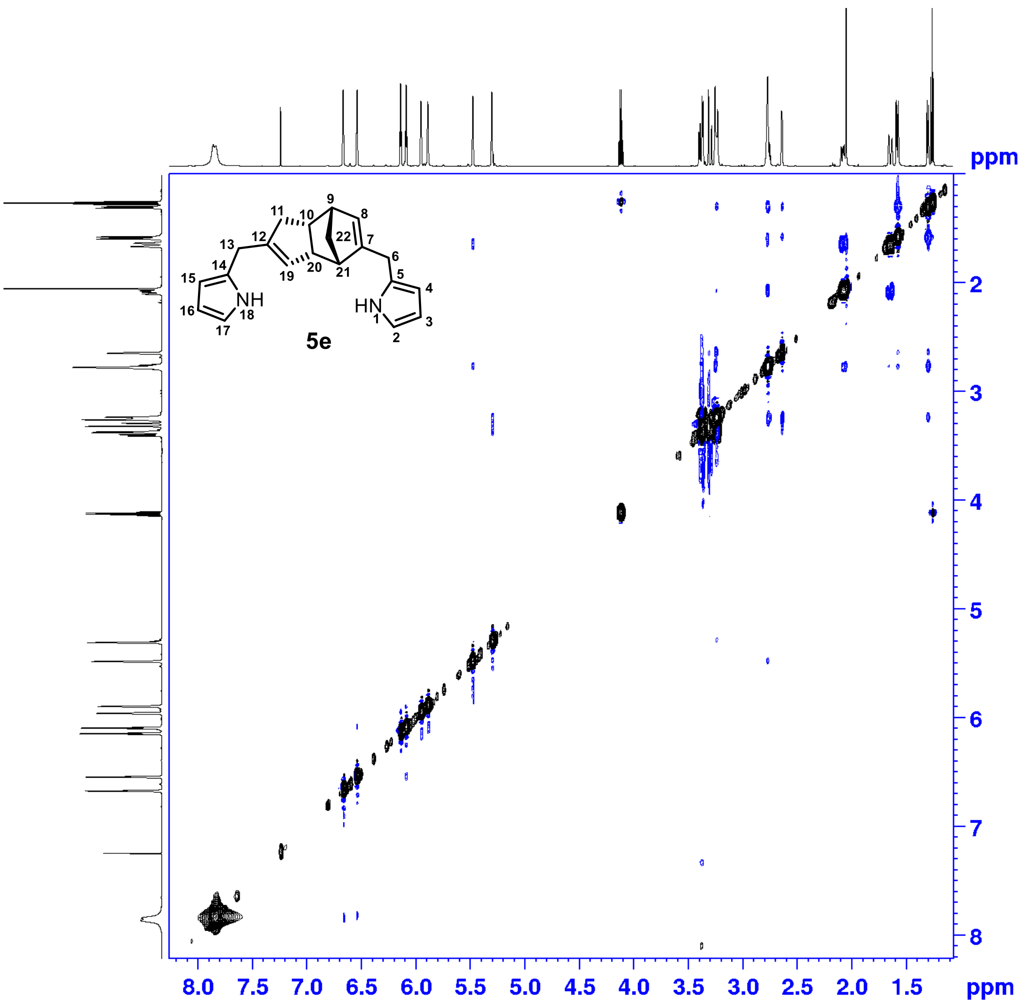

Figure S21. The ^{1}H-^{1}H NOESY spectrum of **5e** ($CDCl_3$, 300 K).

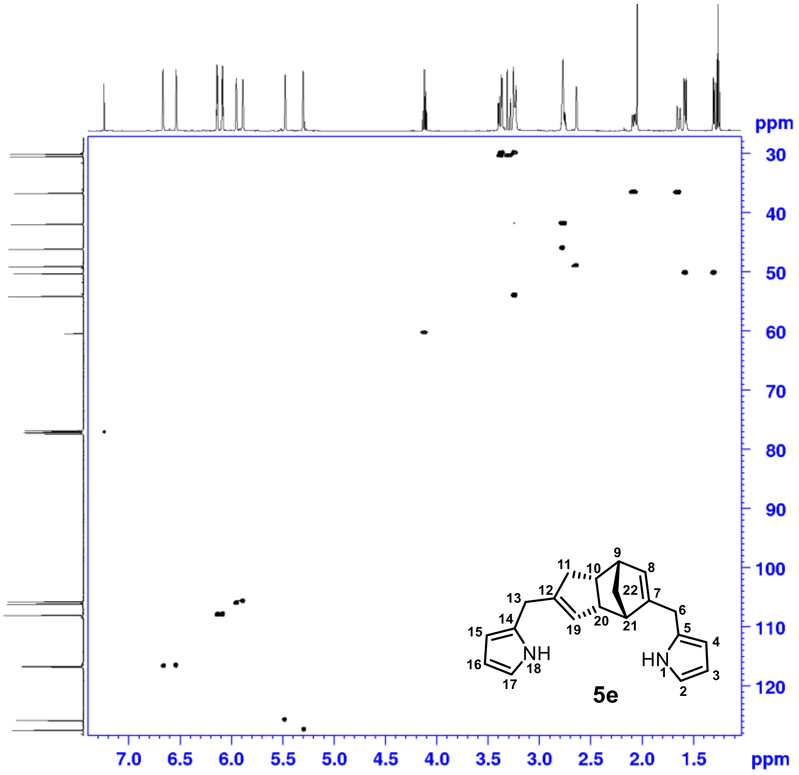

Figure S22. The ^{1}H-^{13}C HSQC spectrum of **5e** ($CDCl_3$, 300 K).

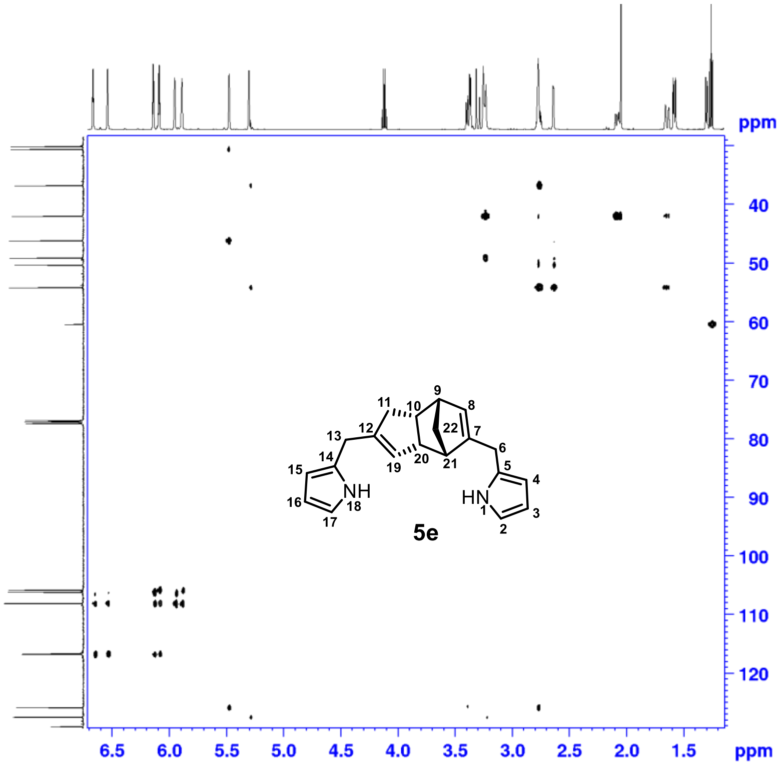

Figure S23. The ^{1}H-^{13}C H2BC spectrum of **5e** ($CDCl_3$, 300 K).

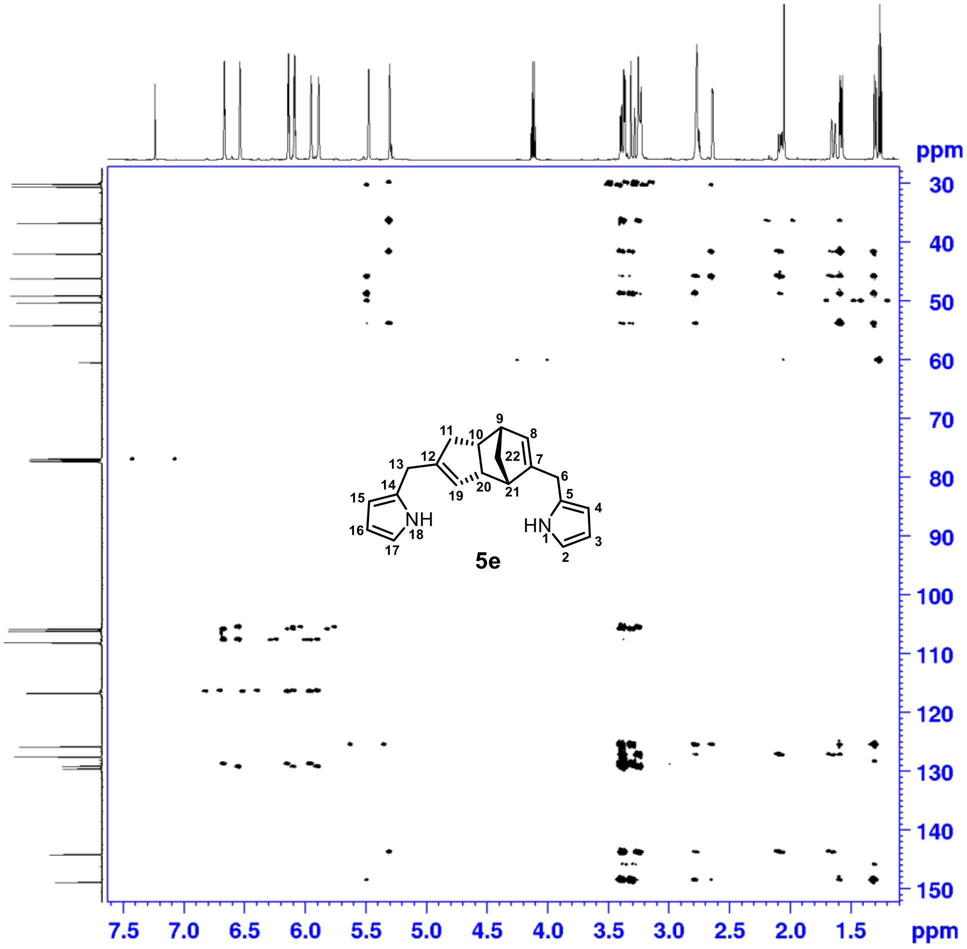

Figure S24. The ^{1}H-^{13}C HMBC spectrum of **5e** ($CDCl_3$, 300 K).

NMR of 3

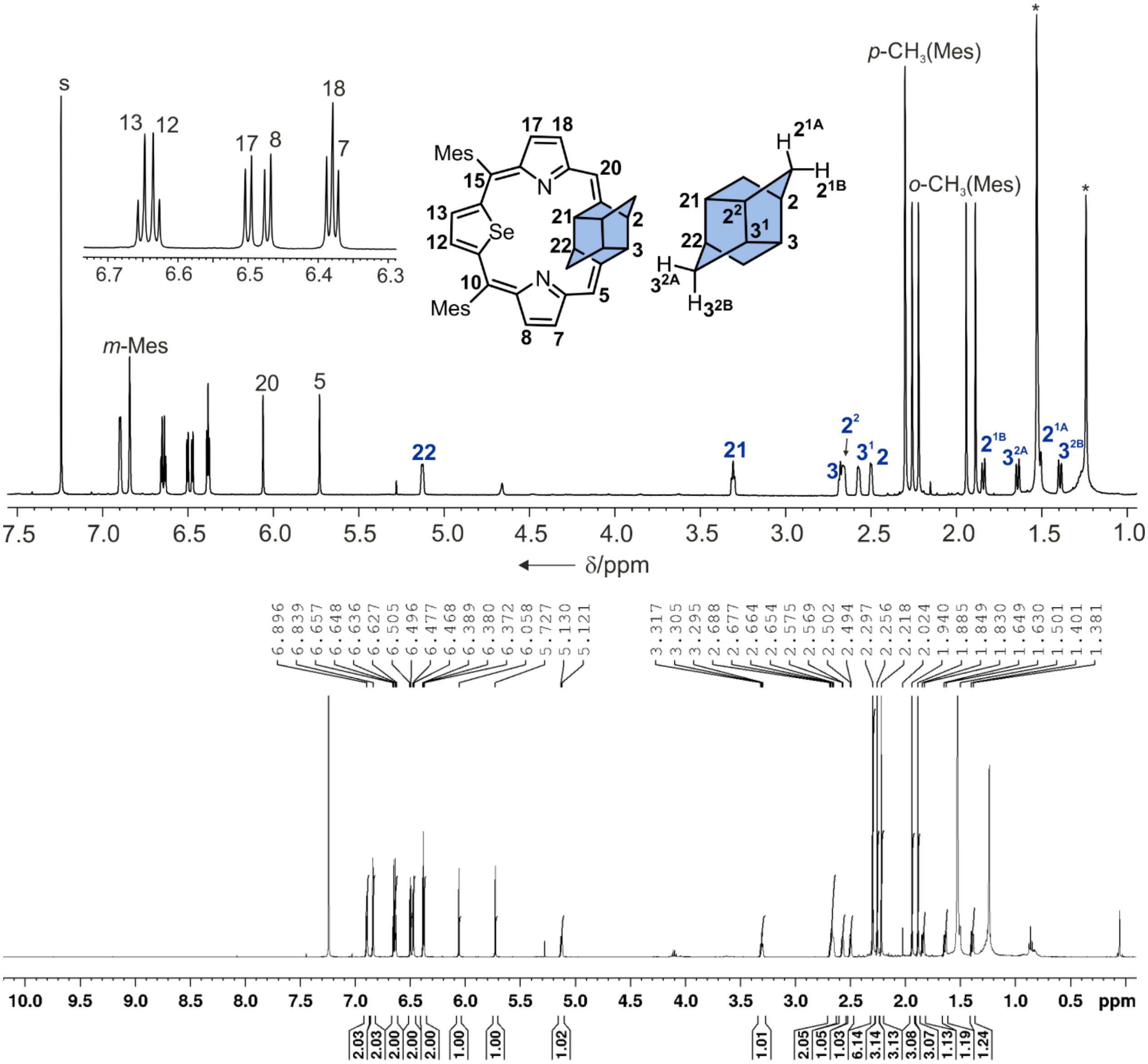

Figure S25. The ^{1}H NMR spectrum of **3** ($CDCl_3$, 300 K), top: peak assignments.

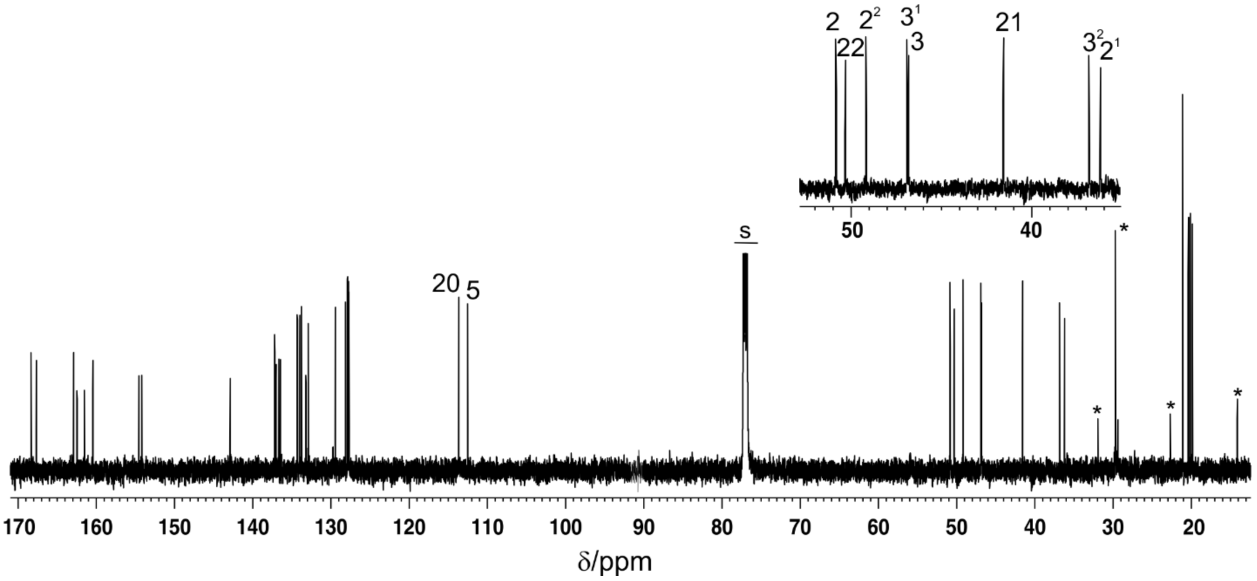

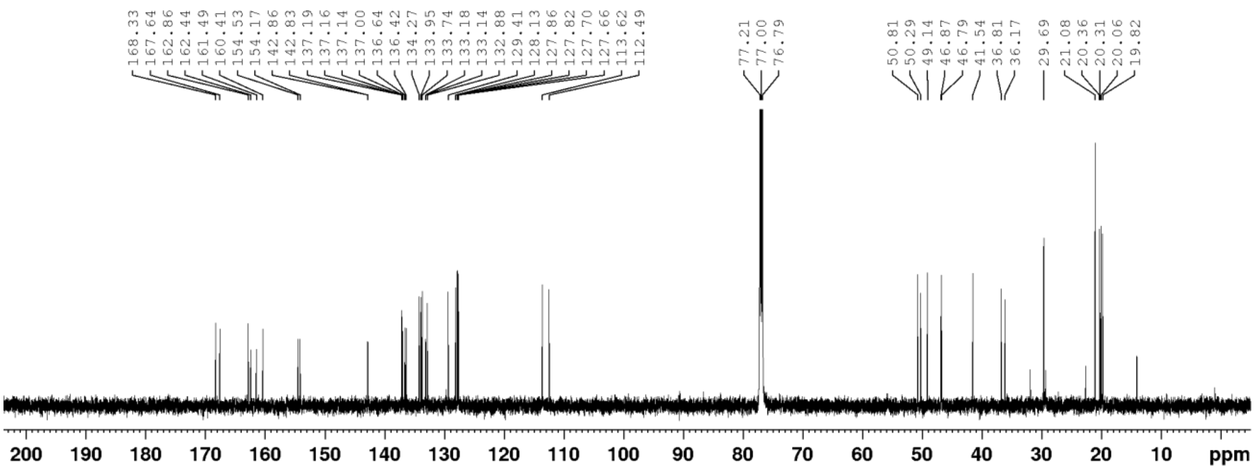

Figure S26. The ^{13}C NMR spectrum of **3** ($CDCl_3$, 300 K), top: peak assignments.

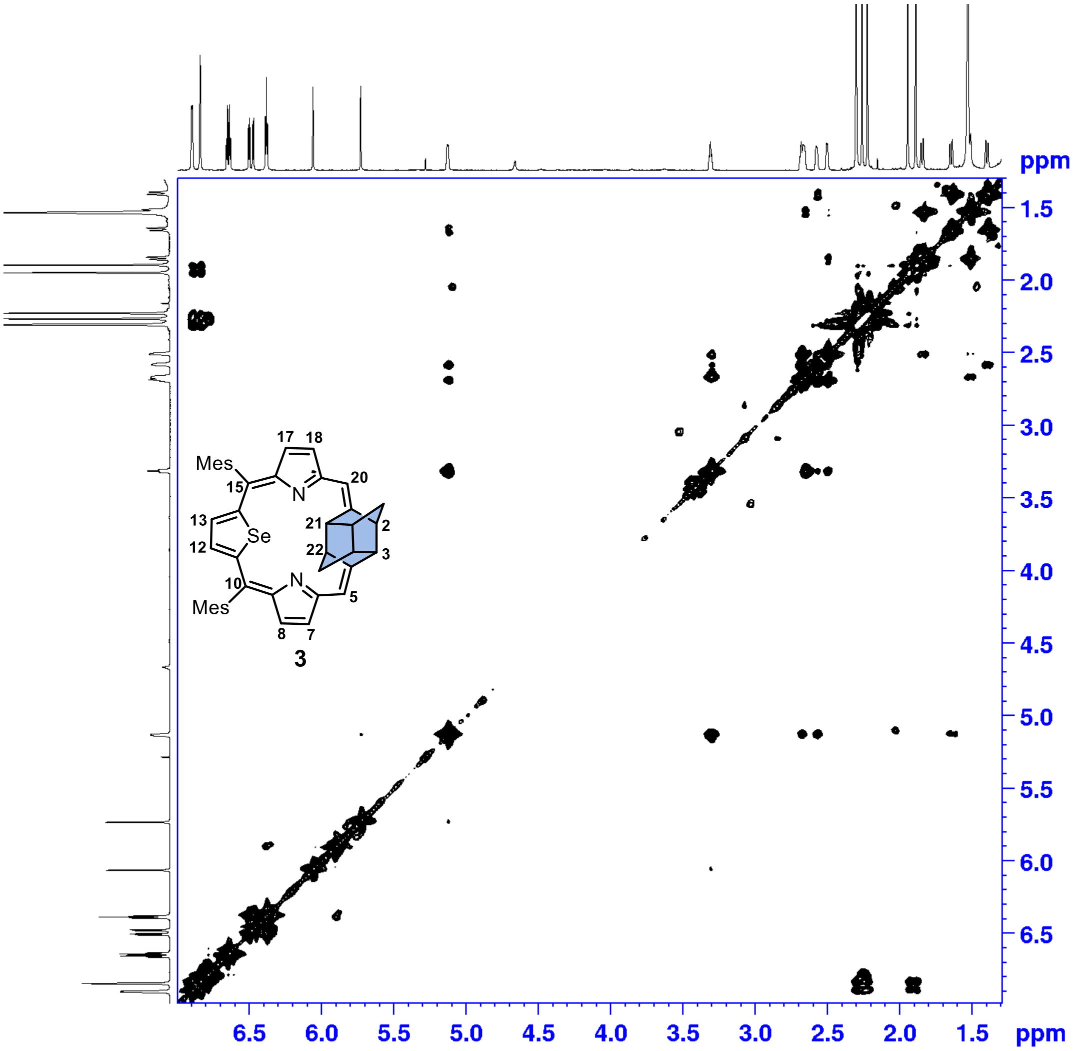

Figure S27. The ^1H-^1H COSY spectrum of **3** ($CDCl_3$, 300 K).

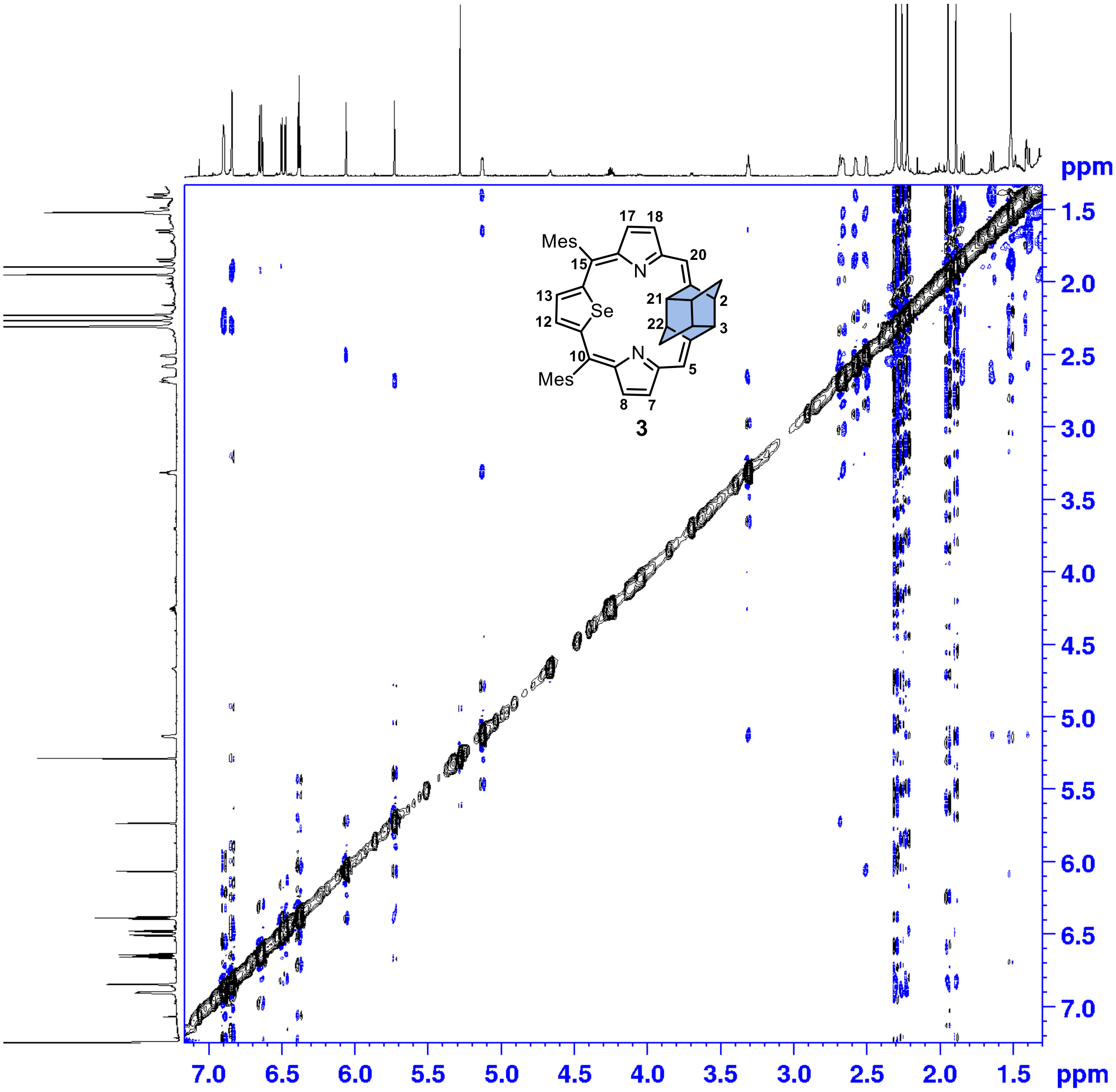

Figure S28. The ^1H-^1H NOESY spectrum of **3** ($CDCl_3$, 300 K).

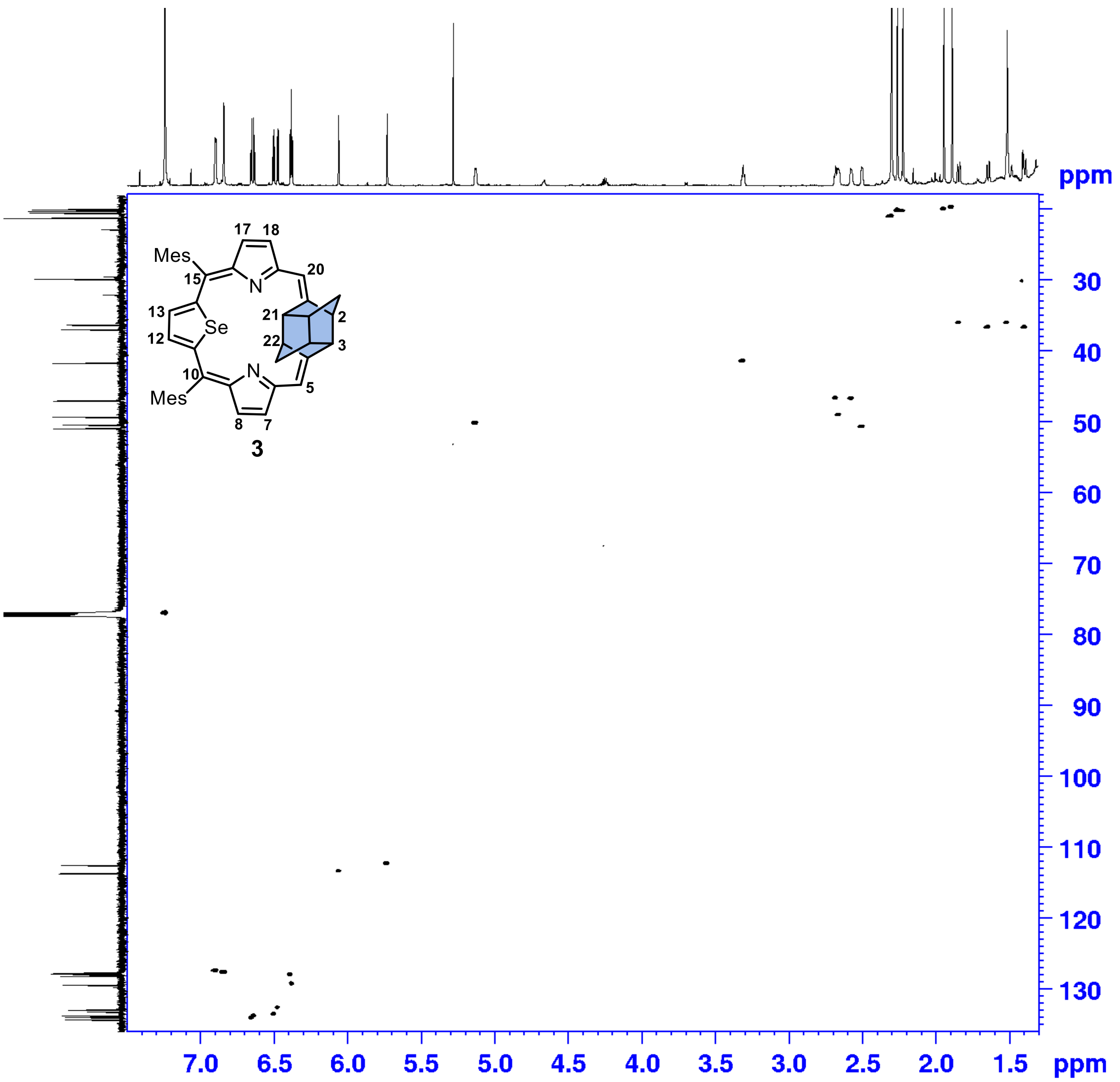

Figure S29. The ^{1}H-^{13}C HSQC spectrum of **3** ($CDCl_3$, 300 K).

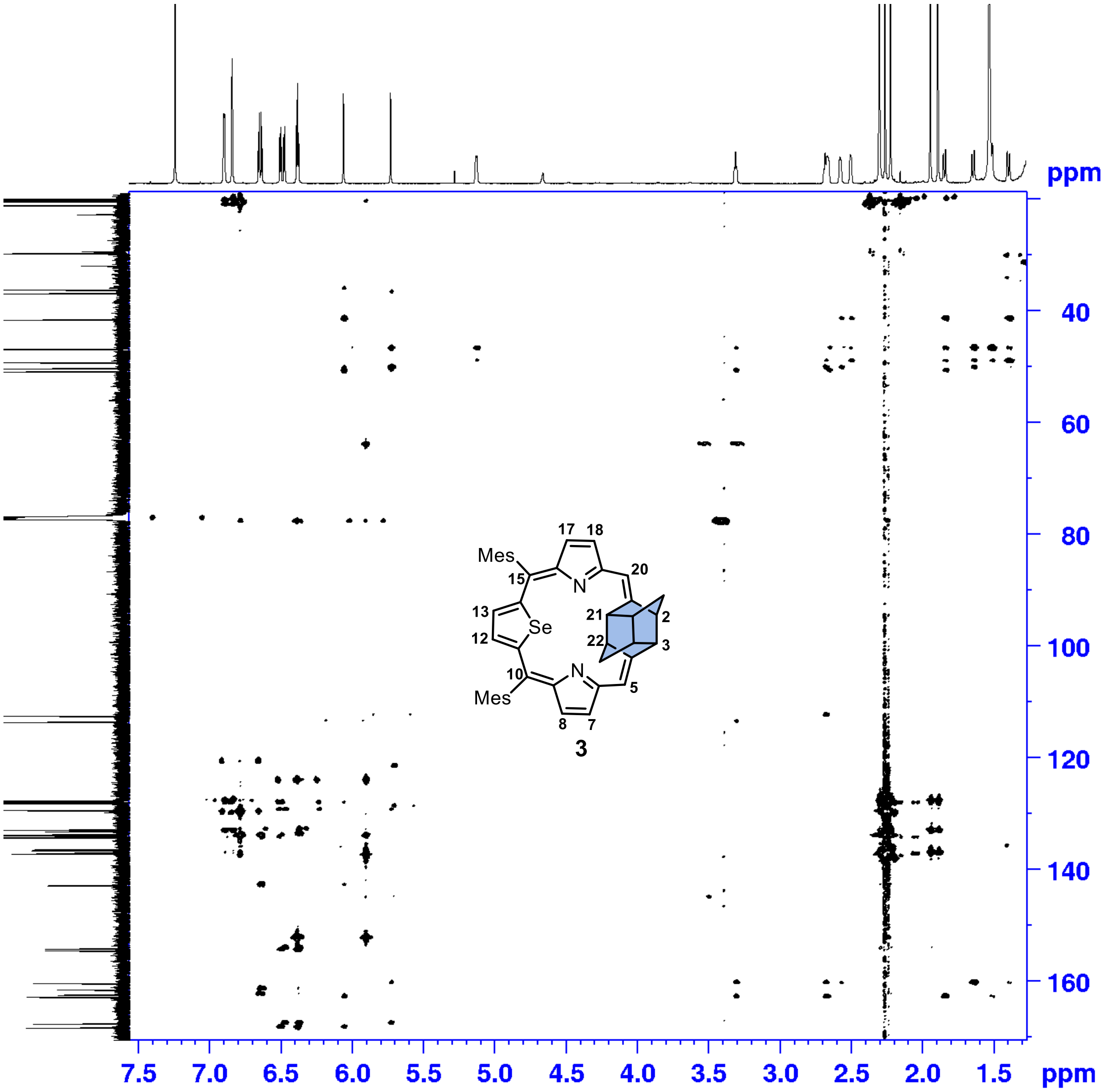

Figure S30. The ^{1}H-^{13}C HMBC spectrum of **3** ($CDCl_3$, 300 K).

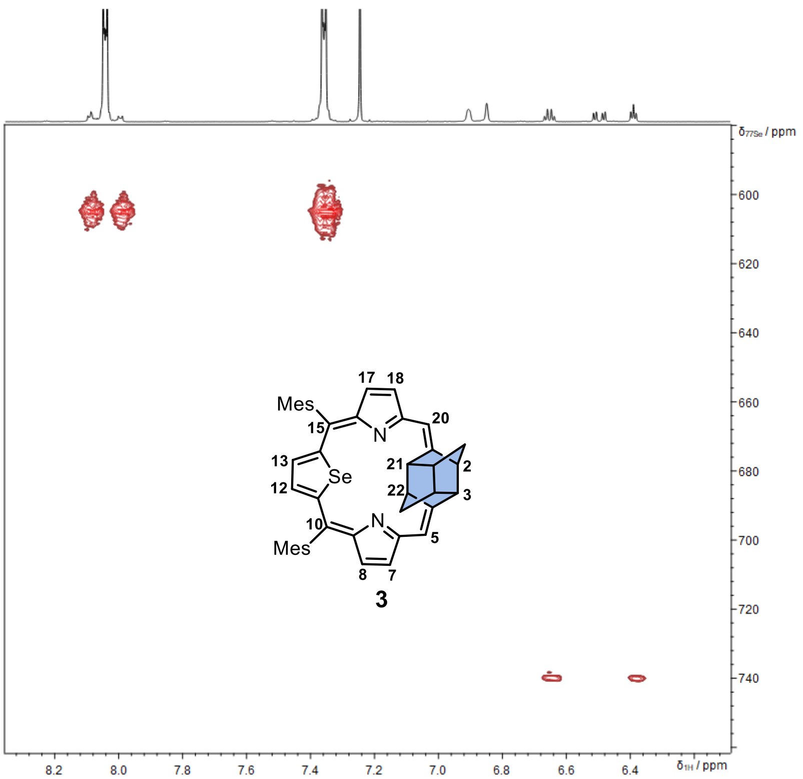

Figure S31. The ^{1}H-^{77}Se HMBC spectrum of **3** ($CDCl_3$, 300 K).

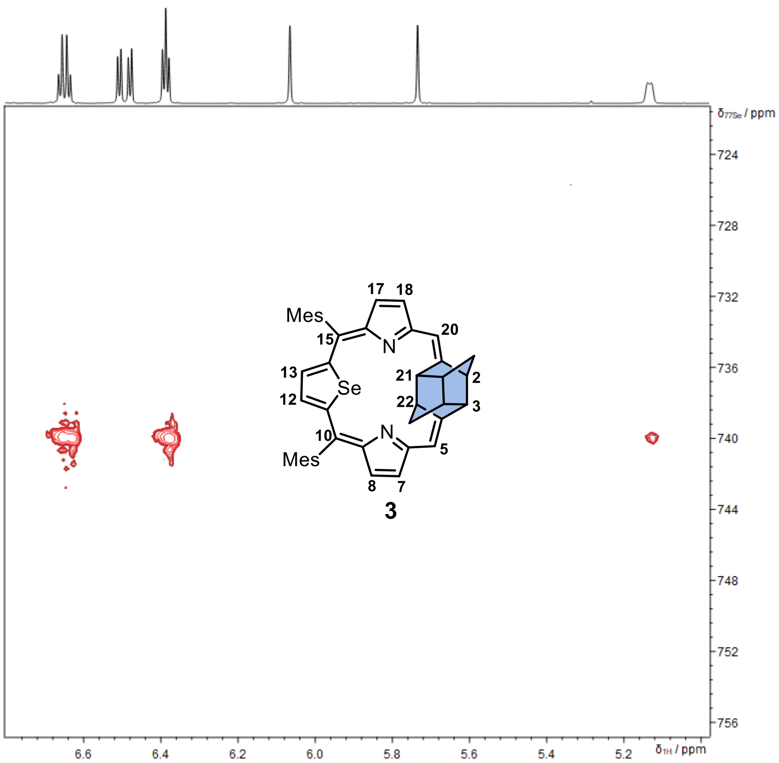

Figure S32. The ^{1}H-^{77}Se HMQC spectrum of **3** ($CDCl_3$, 300 K).

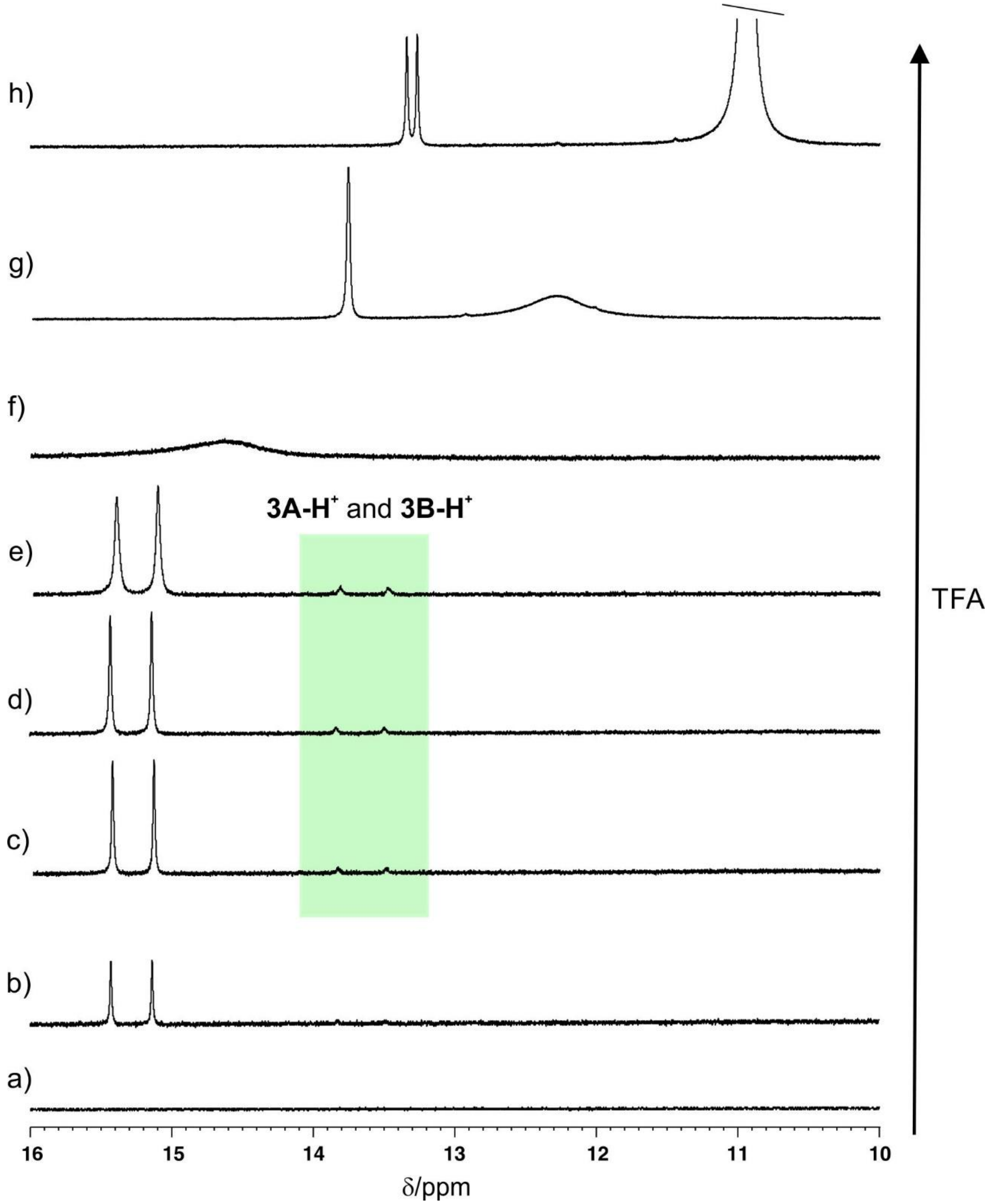

Figure S33. Titration of **3** with TFA (CD_2Cl_2, 180 K). The 16 – 10 ppm range.

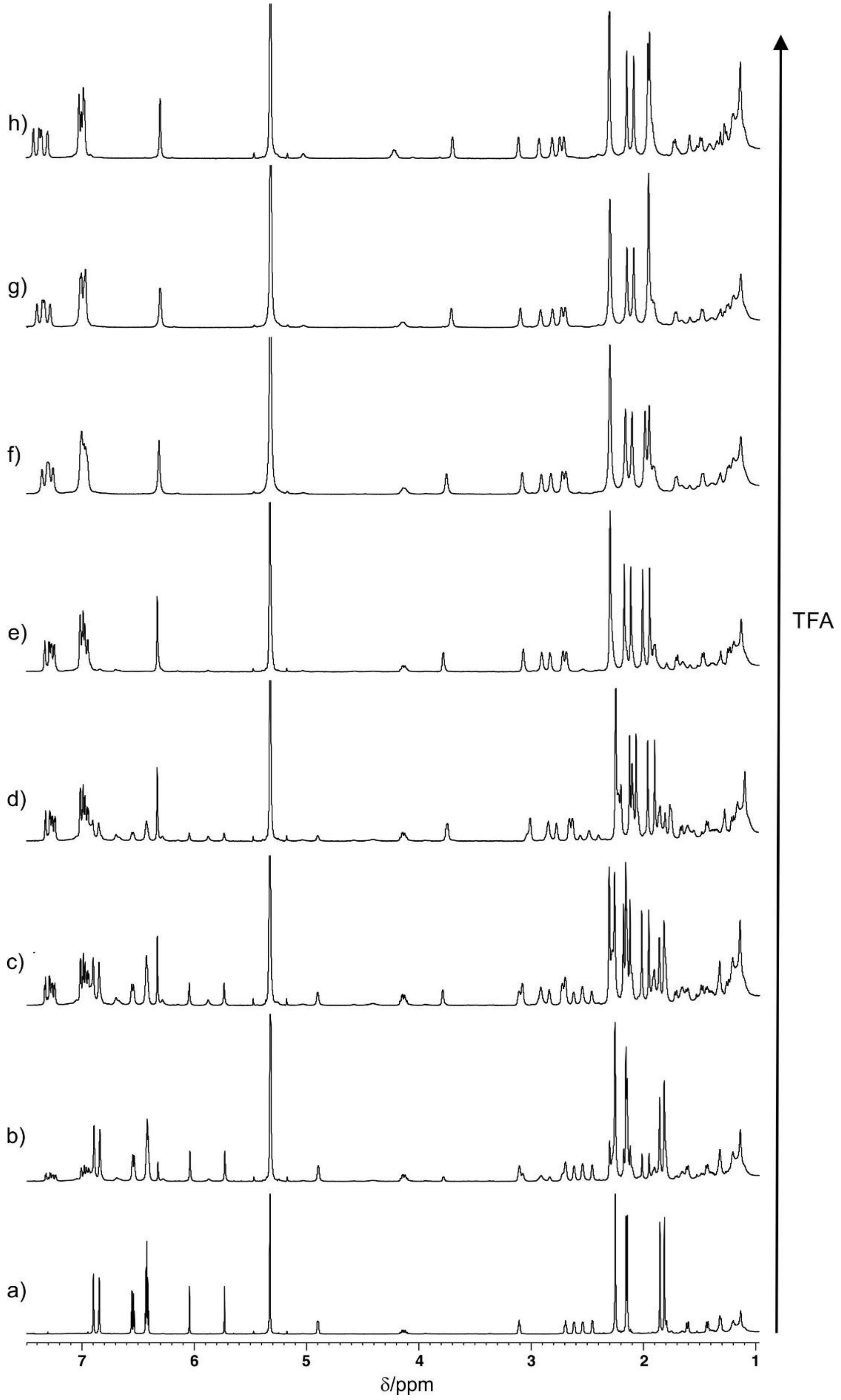

Figure S34. Titration of **3** with TFA (CD_2Cl_2, 180 K). The 7.5 – 1 ppm range.

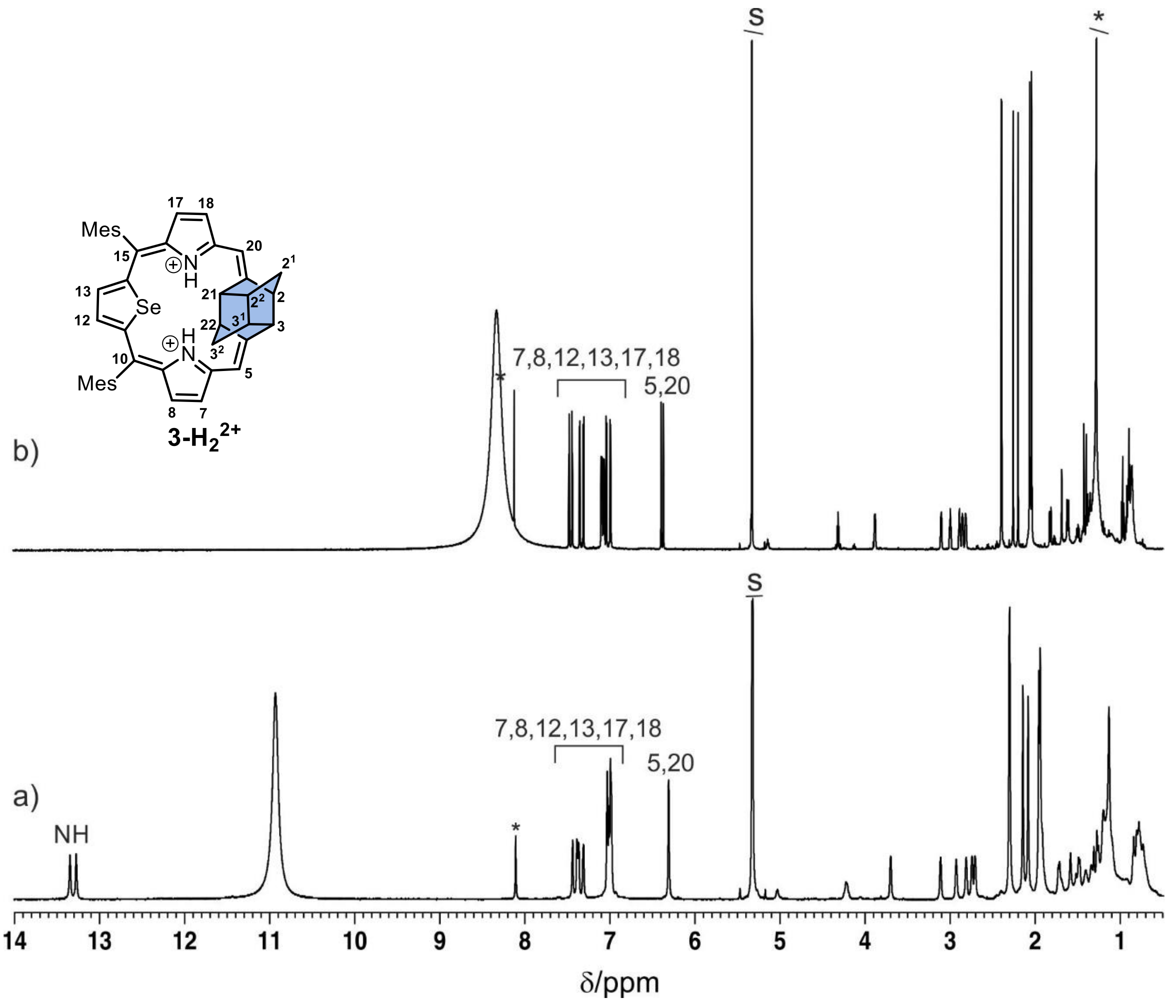

Figure S35. The ^{1}H NMR spectra of **3-H_2^{2+}** (CD_2Cl_2): a) 180 K, b) 300 K.

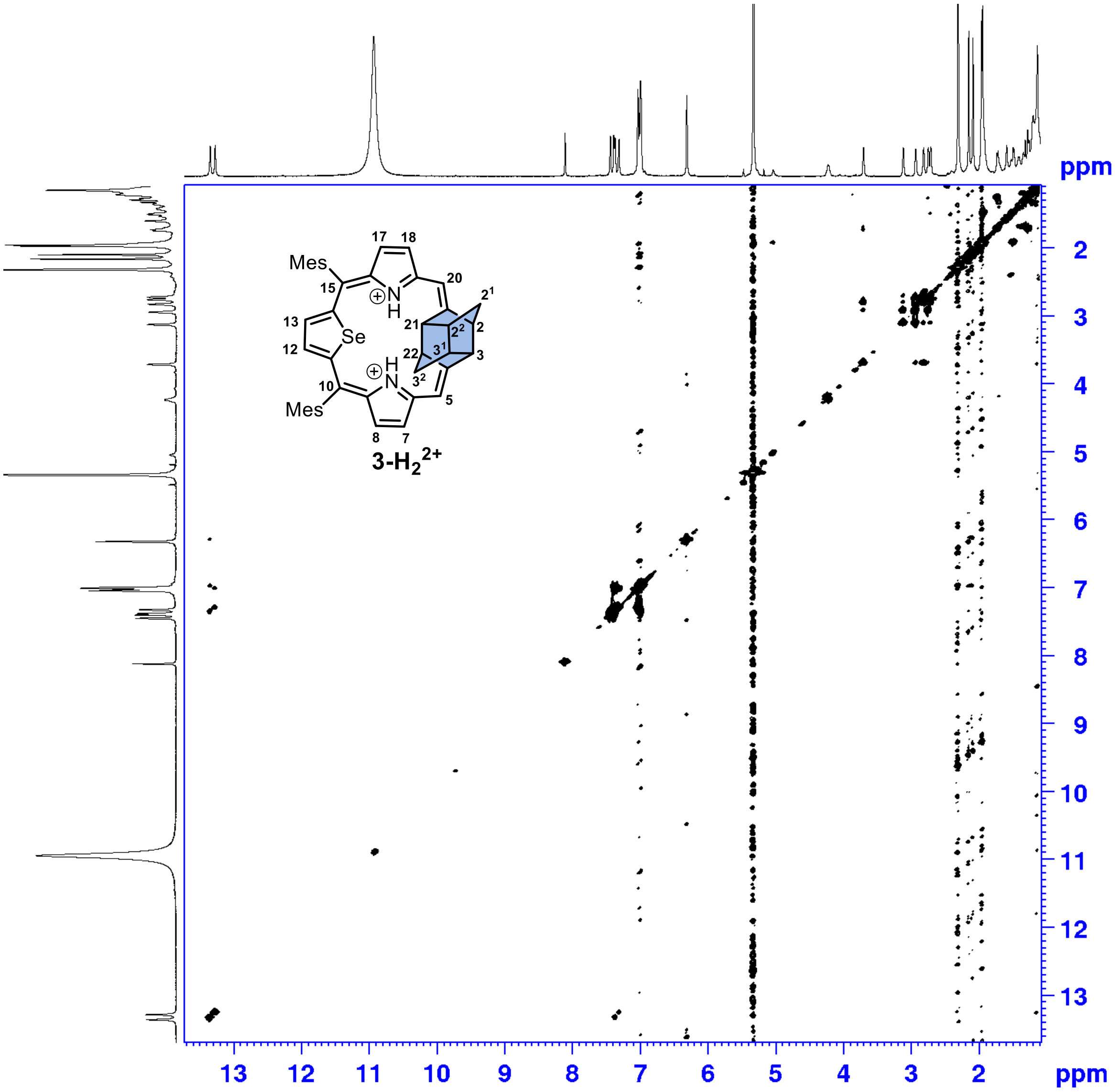

Figure S36. The ^{1}H-^{1}H COSY spectrum of **3-H_2^{2+}** (CD_2Cl_2, 180 K).

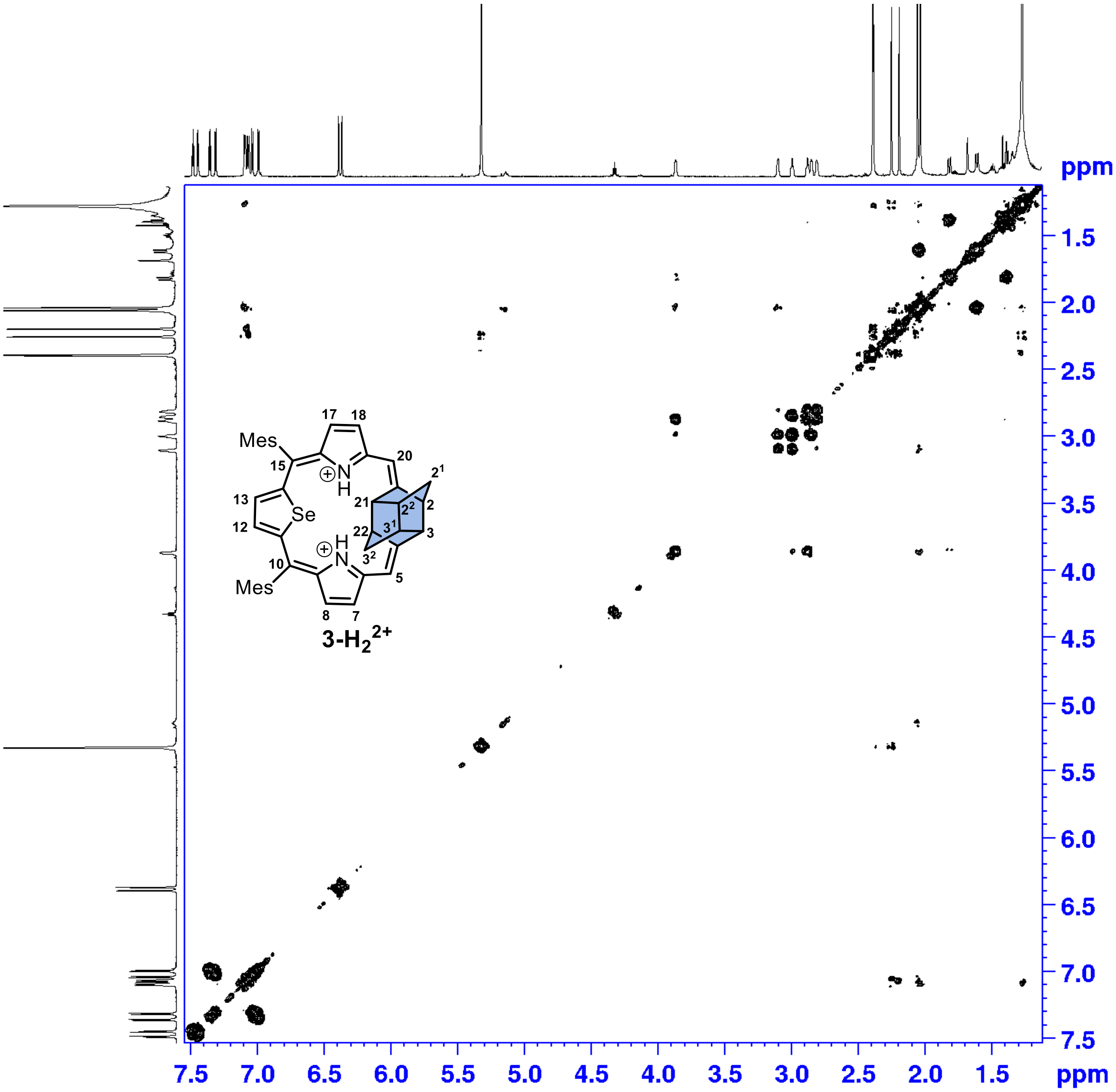

Figure S37. The ^{1}H-^{1}H COSY spectrum of **3-H_2^{2+}** (CD_2Cl_2, 300 K).

6. UV-Vis spectra

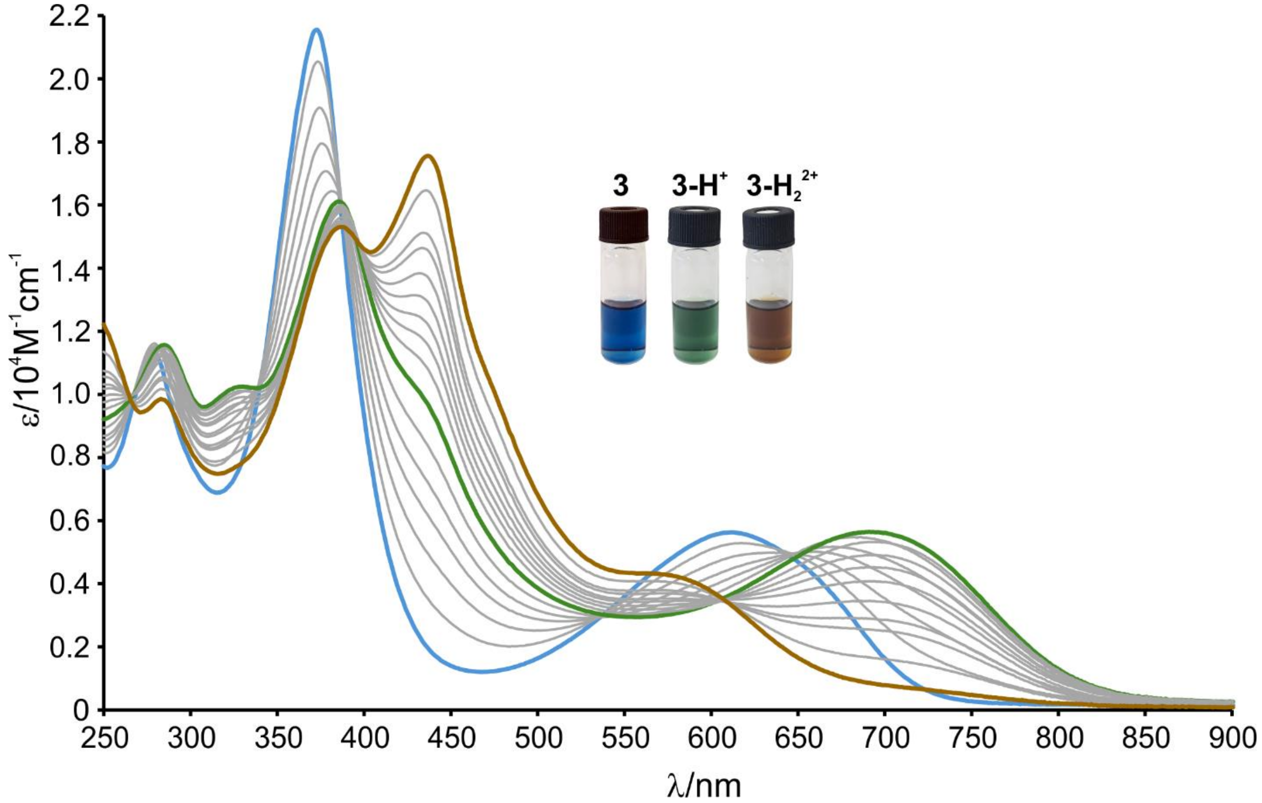

Figure S38. UV/Vis titration of **3** with TFA (CH_2Cl_2, 300 K).

7. MS spectra

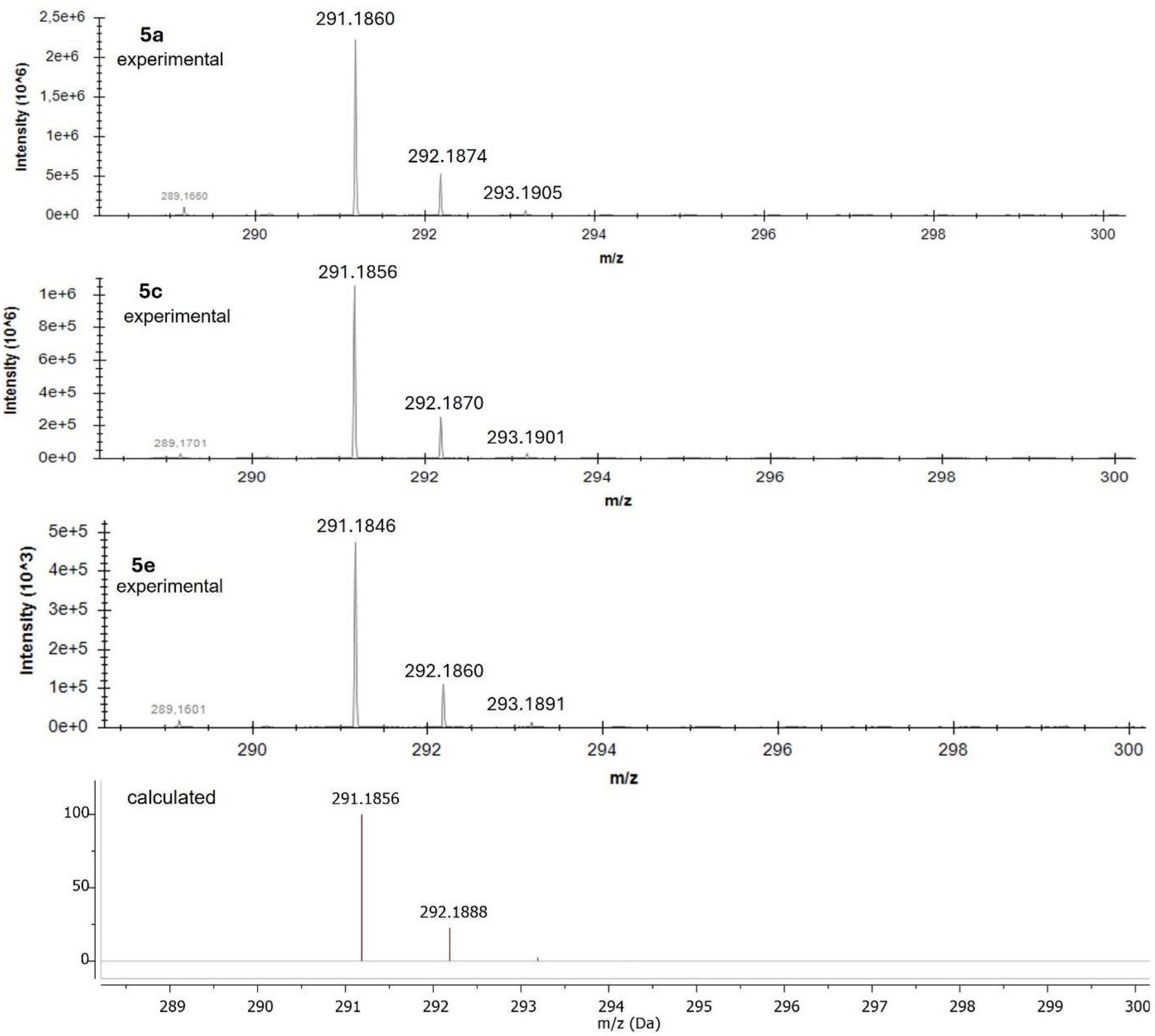

Figure S39. The HR-MS spectra of **5a**, **5b,** and **5c**.

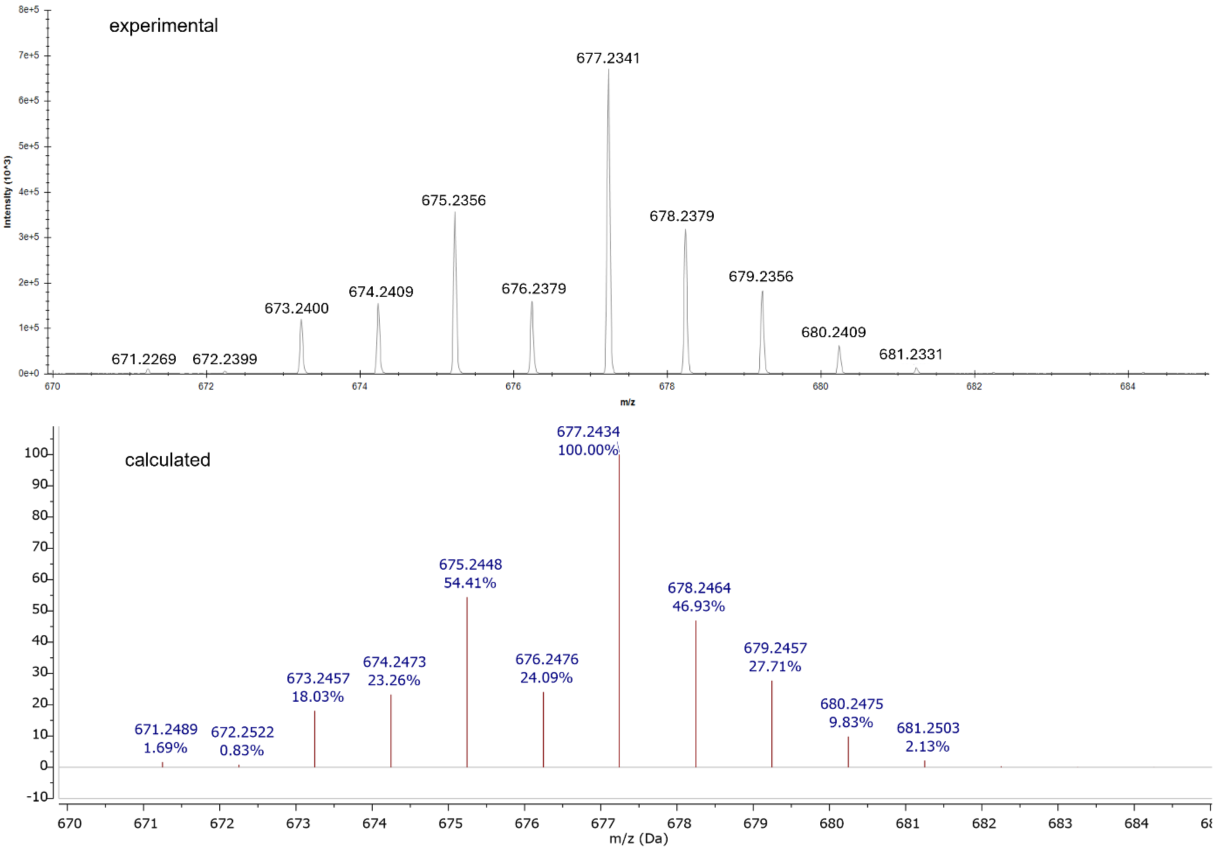

Figure S40. The HR-MS spectra of **3** (*m/z* calcd for $C_{44}H_{41}N_2Se^+$ $[M+H]^+$: 677.2434).

8. DFT calculations

8.1. Optimized structures

The DFT-optimized structures are available at https://zenodo.org/records/19683562.

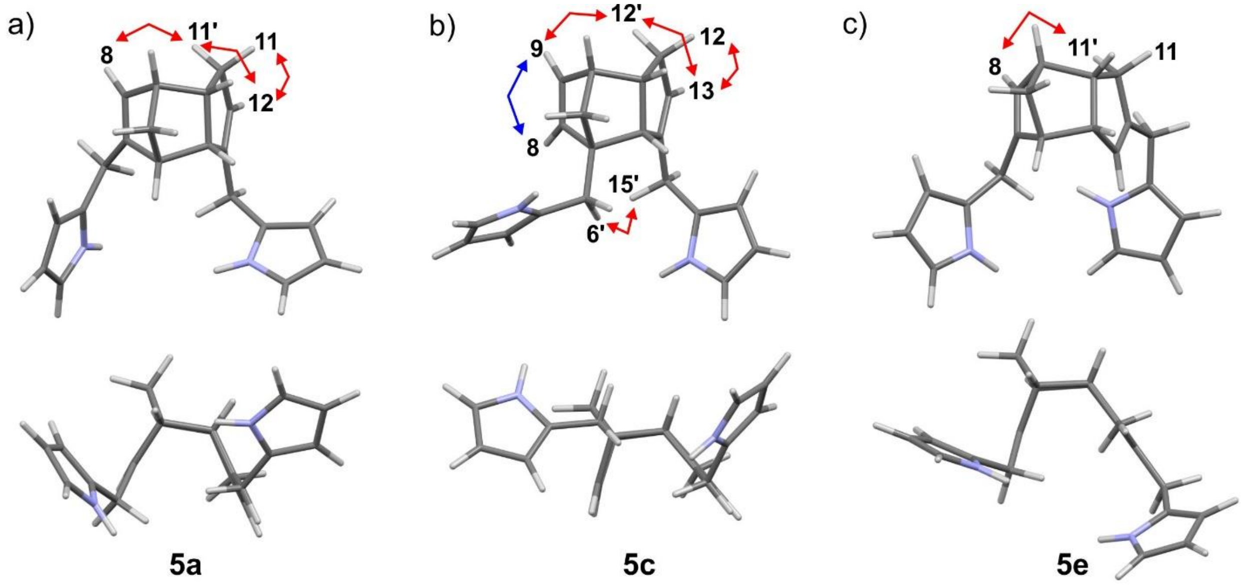

Figure S41. DFT-optimized structures of cycloadducts (B3LYP/6-31G(d,p)): a) **5a**, b) **5c**, and c) **5e** (top: front view and bottom: side view). Red arrows denote the key NOE contacts observed in the NOESY spectrum, while blue arrows indicate crucial correlations in the COSY spectrum for **5c**.

a)

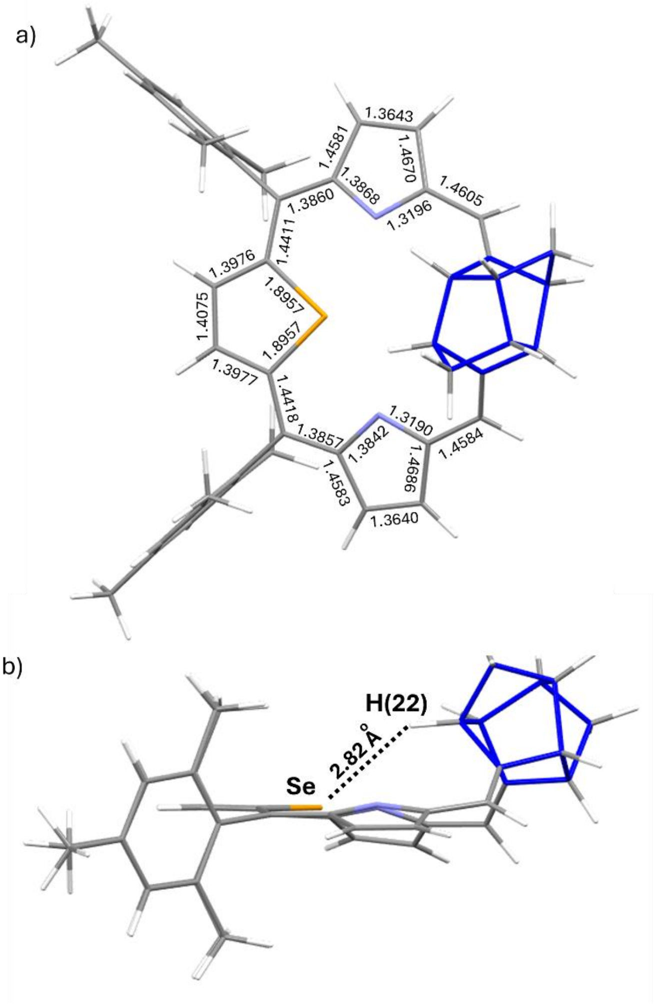

c)

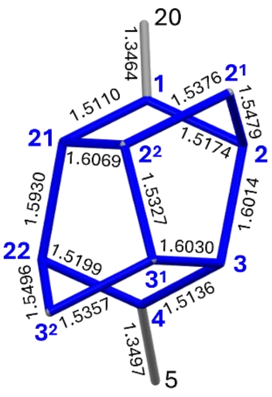

Bond angles (°)

**20–1–21**	130.5	**1–2–2^1**	97.0
**20–1–2**	126.3	**3–2–2^1**	102.3
**21–1–2**	101.6	**2–3–3^1**	103.9
**5–4–22**	130.0	**4–3–3^1**	100.2
**5–4–3**	126.9	**4–3–2**	109.9
**22–4–3**	101.4	**2–2^1–2^2**	93.9
**4–22–21**	107.5	**3–3^1–3^2**	105.4
**4–22–3^2**	96.3	**3–3^1–2^2**	98.7
**21–22–3^2**	102.4	**2^2–3^1–3^2**	102.5
**1–21–22**	110.1	**21–2^2–3^1**	106.0
**1–21–2^2**	99.9	**2^1–2^2–3^1**	102.4
**22–21–2^2**	104.2	**21–2^2–2^1**	98.7
**1–2–3**	107.4	**22–3^2–3^1**	94.4

Figure S42. DFT-optimized structure (B3LYP/6-31G(d,p)) of **3** (enantiomer A): a) front view**,** b) side view, and c) the cage with bond distances. In the table, the bond angles for the cage are involved.

8.2. Correlation between calculated and experimental NMR values

Table S2 ^{1}H and ^{13}C NMR (selected) chemical shifts calculated for **13** using the GIAO method.

Position	^{1}H NMR		^{13}C NMR	
	δ_{exp}(ppm) 300 K, $CDCl_3$	δ_{calc}(ppm)[a]	δ_{exp}(ppm) 300 K, $CDCl_3$	δ_{calc}(ppm)[a]
**2**	2.50	2.22	50.8	53.8
**2^{1A}**	1.51	1.69	36.2	37.6
**2^{1B}**	1.84	1.96		
**2^{2}**	2.68	2.66	49.1	53.0
**3**	2.68	2.68	46.8	50.9
**3^{1}**	2.57	2.52	46.9	50.0
**3^{2A}**	1.64	1.54	36.6	37.8
**3^{2B}**	1.39	1.43		
**5**	5.73	5.68	112.5	110.5
**7**	6.37	6.30	129.4	124.2
**8**	6.47	6.58	132.9	127.6
**12**	6.63	6.49	133.9	126.7
**13**	6.65	6.53	134.3	127.0
**17**	6.50	6.59	133.7	128.3
**18**	6.38	6.37	128.1	123.0
**20**	6.06	6.11	113.6	111.8
**21**	3.31	3.14	41.5	44.8
**22**	5.13	4.95	50.3	54.3

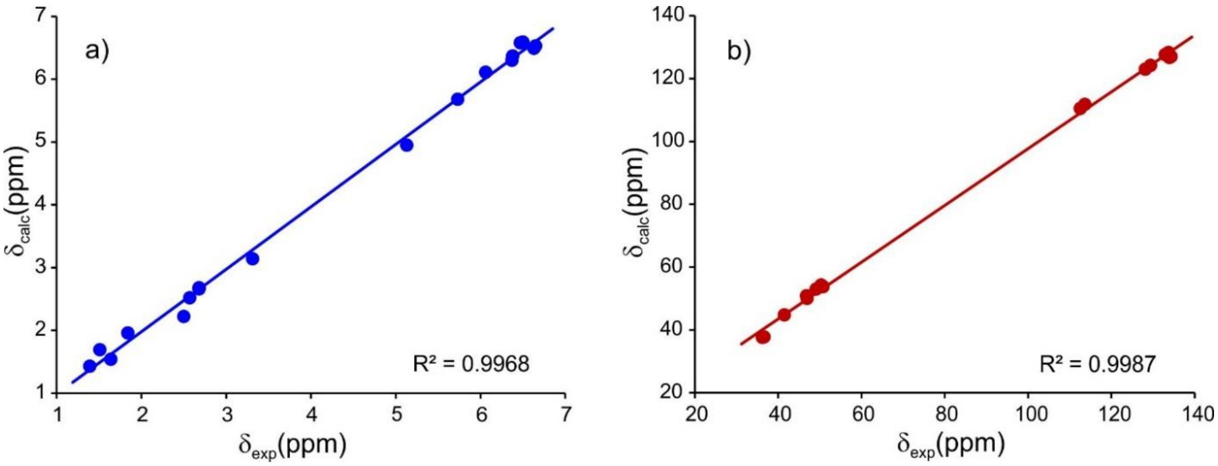

Figure S43. Linear correlation between selected calculated and experimental values of ^{1}H (a) and ^{13}C (b) chemical shifts for **3**.

8.3. Cartesian coordinates

Compound 3

C	3.26271	-2.64128	-1.33812
C	3.47948	-1.23224	-1.02096
C	4.64859	-0.41798	-1.37073
C	4.39973	0.83794	-0.89955
C	3.05833	0.79393	-0.32974
C	2.2151	1.83372	0.02932
C	0.80492	1.60049	0.21297
C	-0.1646	2.55567	0.53083
C	-1.49608	2.1003	0.50344
C	-1.66528	0.75382	0.16878
C	-2.91597	0.06614	-0.03536
C	-2.93294	-1.28245	-0.35374
C	-4.03198	-2.14125	-0.77916
C	-3.47057	-3.34467	-1.09061
C	-2.03452	-3.21939	-0.80999
C	-1.03551	-4.27567	-0.92493
N	2.57465	-0.5042	-0.39437
N	-1.75139	-2.0029	-0.38595
C	-4.19669	0.84135	0.04258
C	-6.59697	2.31525	0.19553
C	-5.87787	2.23418	-0.99995
C	-4.68595	1.50933	-1.09938
C	-7.8975	3.08002	0.26999
C	2.74191	3.23416	0.12574
C	3.27677	3.69634	1.34612
C	3.76099	5.00625	1.41979
C	3.7295	5.86928	0.32114
C	4.22792	7.29069	0.43482
H	5.53521	-0.76867	-1.88408
H	5.02877	1.71478	-0.97419
H	-2.33829	2.7541	0.70637
H	-5.07159	-1.85156	-0.85237
H	-3.97105	-4.23436	-1.45221
H	-6.25134	2.75067	-1.88155
H	-8.06857	3.48271	1.27285
H	-7.91265	3.91427	-0.4378
H	-8.75173	2.43467	0.02932
H	4.1736	5.35954	2.36232
H	3.42266	7.97036	0.74091
H	5.02508	7.37721	1.17925
H	4.61484	7.65704	-0.52082
C	3.19869	5.38725	-0.87834
C	2.7009	4.08572	-0.99817
H	3.17033	6.04025	-1.74795
C	-4.90265	0.91135	1.2615
C	-6.08949	1.64926	1.31442
H	-6.6289	1.70605	2.25742
H	0.09299	3.58578	0.75532
Se	0.00875	-0.08735	-0.12034
C	2.15494	3.6052	-2.32366
C	3.33553	2.79941	2.56202
C	-4.39527	0.20596	2.4989
C	-3.9522	1.43806	-2.41935
H	2.79905	2.83534	-2.76339
H	2.08156	4.43012	-3.03707
H	1.16235	3.15839	-2.212
H	2.34731	2.401	2.81486
H	3.71424	3.3445	3.43051
H	3.98859	1.9367	2.39217
H	-3.37	0.50531	2.74031
H	-4.38209	-0.88071	2.36223
H	-5.0271	0.43032	3.36221
H	-3.98353	0.42546	-2.83719
H	-2.89535	1.69897	-2.30739
H	-4.39785	2.11739	-3.15064
H	-1.28911	-5.10016	-1.59048

H	3.67247	-2.98116	-2.28951
C	2.62748	-3.54154	-0.56417
C	0.11903	-4.35781	-0.23058
C	0.59662	-3.55706	0.96977
C	2.18368	-3.45541	0.87757
C	2.50574	-5.03029	-0.83106
C	1.10016	-5.51027	-0.23226
C	3.50495	-5.4985	0.25449
H	3.58533	-6.58762	0.34729
H	4.5038	-5.07875	0.10683
H	2.68713	-5.34651	-1.85991
H	0.10355	-2.60652	1.14403
C	0.39495	-4.68238	2.01587
C	1.37812	-5.64954	1.34026
H	-0.64099	-5.02973	2.0601
C	2.70185	-4.87999	1.41068
H	3.1791	-4.8564	2.39317
H	1.41051	-6.67691	1.71113
H	0.71943	-4.40508	3.0253
H	0.74189	-6.42008	-0.71861
H	2.56079	-2.5874	1.4167

Compound 5a

C	-1.4325	2.6614	-0.0652
C	-1.4551	1.3190	-0.0845
C	-0.4900	0.8446	1.0007
C	0.9824	1.1716	0.5304
C	1.0015	2.7420	0.5187
C	-0.4488	3.1028	1.0058
C	1.3825	0.7731	-0.8699
C	1.5954	1.8454	-1.6433
C	1.4091	3.1586	-0.9209
C	-0.6719	1.9738	2.0441
C	1.5191	-0.6735	-1.2869
C	2.4735	-1.4697	-0.4364
C	-2.1510	0.4329	-1.0816
C	-2.8452	-0.7614	-0.4852
C	-3.5631	-0.9223	0.6844
C	-4.0381	-2.2653	0.7270
C	-3.6006	-2.8936	-0.4174
N	-2.8735	-1.9750	-1.1411
N	2.0240	-2.3511	0.5242
C	3.0880	-2.9279	1.1786
C	4.2411	-2.4080	0.6328
C	3.8542	-1.4880	-0.3832
H	-1.9417	3.3232	-0.7588
H	-0.6393	-0.1800	1.3470
H	1.6763	0.7507	1.2685
H	1.7209	3.1280	1.2476
H	-0.5563	4.1315	1.3586
H	1.8905	1.7955	-2.6893
H	0.6488	3.7853	-1.4064
H	2.3358	3.7483	-0.9254
H	0.0805	1.9549	2.8412
H	-1.6730	1.9922	2.4843
H	1.8346	-0.7180	-2.3355
H	0.5338	-1.1622	-1.2430
H	-1.4100	0.0760	-1.8134
H	-2.8582	1.0465	-1.6576
H	-3.7275	-0.1517	1.4245
H	-4.6295	-2.7184	1.5103
H	-3.7371	-3.9042	-0.7722
H	-2.4554	-2.1464	-2.0418
H	1.0526	-2.5443	0.7115
H	2.9357	-3.6554	1.9617
H	5.2499	-2.6628	0.9263
H	4.5106	-0.8987	-1.0085

Compound 5c

C	1.3190	2.6964	0.7601
C	1.2296	1.3914	1.0463
C	0.7991	0.6575	-0.2182
C	-0.7088	1.0847	-0.5012
C	-0.5887	2.6090	-0.8606
C	0.9494	2.8743	-0.7063
C	-1.6991	1.0506	0.6454
C	-2.1371	2.2781	0.9524
C	-1.5460	3.3675	0.0951
C	1.5023	1.5502	-1.2794
C	-2.1372	-0.2182	1.3352
C	-2.7666	-1.2274	0.4111
C	1.0535	-0.8580	-0.2813
C	2.4653	-1.2953	-0.0236
C	3.0566	-1.8243	1.1084
C	4.4327	-2.0576	0.8224
C	4.6506	-1.6681	-0.4798
N	3.4542	-1.2081	-0.9820
N	-2.5368	-2.5799	0.5501
C	-3.2593	-3.2865	-0.3848
C	-3.9628	-2.3746	-1.1395
C	-3.6527	-1.0773	-0.6386
H	1.5182	3.5047	1.4552
H	1.3466	0.9238	2.0179
H	-1.0738	0.4830	-1.3436
H	-0.8677	2.7855	-1.9043
H	1.2894	3.8004	-1.1767
H	-2.8562	2.4862	1.7416
H	-1.0219	4.1203	0.6999
H	-2.3262	3.9108	-0.4547
H	1.1677	1.3439	-2.3048
H	2.5928	1.4876	-1.2226
H	-2.8317	0.0449	2.1448
H	-1.2718	-0.6856	1.8283
H	0.4144	-1.3621	0.4507
H	0.7162	-1.2236	-1.2618
H	2.5474	-2.0246	2.0416
H	5.1746	-2.4687	1.4924
H	5.5430	-1.6838	-1.0875
H	3.3128	-0.8656	-1.9186
H	-1.9316	-2.9889	1.2441
H	-3.2055	-4.3636	-0.4360
H	-4.6232	-2.6105	-1.9622
H	-4.0349	-0.1322	-0.9985

Compound 5e

C	-1.0570	1.8461	-1.3225
C	-1.2920	0.8200	-0.4893
C	-1.3327	1.3940	0.9225
C	0.1401	1.8282	1.3019
C	0.4084	3.0344	0.3277
C	-0.9323	3.1120	-0.4874
C	1.2542	0.8638	1.0051
C	2.0875	1.3021	0.0527
C	1.7041	2.6829	-0.4455
C	-1.9464	2.7856	0.6386
C	3.3062	0.5961	-0.5000
C	3.3984	-0.8702	-0.2026
C	-1.3655	-0.6463	-0.8101
C	-2.6440	-1.3026	-0.3598
C	-3.9653	-0.9301	-0.5173
C	-4.7778	-1.9386	0.0754
C	-3.9342	-2.9026	0.5818
N	-2.6442	-2.5064	0.3119
N	2.5554	-1.7821	-0.8040
C	2.8394	-3.0560	-0.3649
C	3.8786	-2.9641	0.5335
C	4.2304	-1.5862	0.6350
H	-0.8863	1.7826	-2.3927

H	-1.8267	0.7713	1.6713
H	0.1421	2.1366	2.3559
H	0.5321	3.9681	0.8849
H	-1.0712	4.0538	-1.0243
H	1.3617	-0.0910	1.5120
H	1.5596	2.6876	-1.5352
H	2.5070	3.4067	-0.2460
H	-1.8888	3.4711	1.4925
H	-2.9784	2.7301	0.2814
H	3.3401	0.7614	-1.5885
H	4.2118	1.0847	-0.1152
H	-1.2249	-0.7813	-1.8923
H	-0.5194	-1.1564	-0.3247
H	-4.3029	-0.0282	-1.0085
H	-5.8574	-1.9535	0.1270
H	-4.1384	-3.8224	1.1089
H	-1.8143	-3.0112	0.5796
H	1.8348	-1.5400	-1.4652
H	2.2908	-3.9102	-0.7332
H	4.3397	-3.7912	1.0546
H	5.0101	-1.1603	1.2516

9. References

(1) C. J. White, T. Wang, R. A. Jacobson, R. J. Angelici, "Synthesis, Equilibrium Binding, and ^{77}Se NMR Studies of η^1-Selenophene (Seln) Complexes: [CpRu(CO)(PPh_3)(η^1(Se)-Seln)]BF_4" *Organometallics* **13**, (1994): 4474.

(2) C. J. White, R. J. Angelici, "Synthesis, Reactions, and ^{77}Se NMR Studies of η^5-Selenophene Complexes of Chromium, Manganese, Ruthenium, and Iridium" *Organometallics* **14**, (1995): 332.

(3) M. J. Frisch, G. W. Trucks, H. B. Schlegel, G. E. Scuseria, M. A. Robb, J. R. Cheeseman, G. Scalmani, V. Barone, G. A. Petersson, H. Nakatsuji, X. Li, M. Caricato, A. V. Marenich, J. Bloino, B. G. Janesko, R. Gomperts, B. Mennucci, H. P. Hratchian, J. V. Ortiz, A. F. Izmaylov, J. L. Sonnenberg, D. Williams-Young, F. Ding, F. Lipparini, F. Egidi, J. Goings, B. Peng, A. Petrone, T. Henderson, D. Ranasinghe, V. G. Zakrzewski, J. Gao, N. Rega, G. Zheng, W. Liang, M. Hada, M. Ehara, K. Toyota, R. Fukuda, J. Hasegawa, M. Ishida, T. Nakajima, Y. Honda, O. Kitao, H. Nakai, T. Vreven, K. Throssell, J. A. Montgomery, Jr., J. E. Peralta, F. Ogliaro, M. J. Bearpark, J. J. Heyd, E. N. Brothers, K. N. Kudin, V. N. Staroverov, T. A. Keith, R. Kobayashi, J. Normand, K. Raghavachari, A. P. Rendell, J. C. Burant, S. S. Iyengar, J. Tomasi, M. Cossi, J. M. Millam, M. Klene, C. Adamo, R. Cammi, J. W. Ochterski, R. L. Martin, K. Morokuma, O. Farkas, J. B. Foresman, and D. J. Fox, Gaussian 16, Revision C.01; Gaussian, Inc.: Wallingford CT, 2016.

(4) C. Lee, W. Yang, R. G. Parr "Development of the Colle-Salvetti correlation-energy formula into a functional of the electron density" *Physical Review B* **37**, (1988): 785.

(5) A. D. Becke " Density-functional exchange-energy approximation with correct asymptotic behavior" *Physical Review A* **38**, (1988): 3098.

(6) Rigaku Oxford Diffraction (2025). CrysAlis PRO Software system, Rigaku Oxford Diffraction, Oxford, UK.

(7) G. M. Sheldrick, "SHELXT-Integrated Space-Group and Crystal-Structure Determination" *Acta Crystallographica Section A* **A71**, (2015): 3−8.

(8) G. M. Sheldrick, "Crystal structure refinement with *SHELXL*" *Acta Crystallographica Section C* **C71**, (2015): 3−8.

(9) O. V. Dolomanov, L. J. Bourhis, R. J. Gildea, J. A. Howard, H. Puschmann, "OLEX2: a complete structure solution, refinement and analysis program" *Journal of Applied Crystallography* **42**, (2009): 339.

(10) W. S. Seo, Y. J. Cho, S. C. Yoon, J. T. Park, Y. Park "Synthesis and structure of *ansa*-cyclopentadienyl pyrrolyl titanium complexes: [(η^5-C_5H_4)CH_2(2-C_4H_3N)]Ti(NMe_2)$_2$ and [1,3-{CH_2(2-C_4H_3N)}$_2$(η^5-C_5H_3)]Ti(NMe_2)" *Journal of Organometallic Chemistry* **640**, (2001): 79.

(11) S. J. Narayanan, B. Sridevi, T. K. Chandrashekar, A. Vij, R. Roy, "Novel Core-Modified Expanded Porphyrins with *meso*-Aryl Substituents: Synthesis, Spectral and Structural Characterization" *Journal of American Chemical Society* **121**, (1999): 9053.

10. Author Contributions

K. Stasiak: synthesis of **3** (leading), NMR and UV/Vis measurements (supporting), data analysis (supporting).

K. Ślepokura: X-ray analysis of **3**, i.e., diffraction data collection; crystal structure determination and deposition of the CIF file at the CCDC; preparation of the crystallographic experimental part in the Supporting Information and manuscript.

A. Szumańska: optimization of synthesis of **3** and **5** (supporting).

M. J. Białek: DFT modeling, analysis, and corrections to a prepared manuscript (supporting).

L. Latos-Grażyński: discussion and corrections of original draft (supporting).

A. Berlicka: synthesis of **3** (supporting), synthesis of **5** (leading), data analysis (leading), NMR and UV/Vis measurements (leading), writing of an original draft, preparation of Supporting Information, project administration, funding acquisition.
